# Non-proteolytic ubiquitin modification of PPARγ by Smurf1 protects the liver from steatosis

**DOI:** 10.1371/journal.pbio.3000091

**Published:** 2018-12-19

**Authors:** Kun Zhu, Yi Tang, Xuan Xu, Hien Dang, Liu-Ya Tang, Xiang Wang, Xin Wei Wang, Ying E. Zhang

**Affiliations:** 1 Laboratory of Cellular and Molecular Biology, Center for Cancer Research, National Cancer Institute, National Institutes of Health, Bethesda, Maryland, United States of America; 2 Laboratory of Human Carcinogenesis, Center for Cancer Research, National Cancer Institute, National Institutes of Health, Bethesda, Maryland, United States of America; 3 Laboratory of Cancer Biology and Genetics, Center for Cancer Research, National Cancer Institute, National Institutes of Health, Bethesda, Maryland, United States of America; Washington University in Saint Louis, UNITED STATES

## Abstract

Nonalcoholic fatty liver disease (NAFLD) is characterized by abnormal accumulation of triglycerides (TG) in the liver and other metabolic syndrome symptoms, but its molecular genetic causes are not completely understood. Here, we show that mice deficient for ubiquitin ligase (E3) Smad ubiquitin regulatory factor 1 (Smurf1) spontaneously develop hepatic steatosis as they age and exhibit the exacerbated phenotype under a high-fat diet (HFD). Our data indicate that loss of Smurf1 up-regulates the expression of peroxisome proliferator-activated receptor γ (PPARγ) and its target genes involved in lipid synthesis and fatty acid uptake. We further show that PPARγ is a direct substrate of Smurf1-mediated non-proteolytic lysine 63 (K63)-linked ubiquitin modification that suppresses its transcriptional activity, and treatment of Smurf1-deficient mice with a PPARγ antagonist, GW9662, completely reversed the lipid accumulation in the liver. Finally, we demonstrate an inverse correlation of low SMURF1 expression to high body mass index (BMI) values in human patients, thus revealing a new role of SMURF1 in NAFLD pathogenesis.

## Abstract 2

Non-proteolytic ubiquitination of peroxisome proliferator-activated receptor γ (PPARγ) by the E3 ligase Smurf1 suppresses its transcriptional activity and plays a critical role in preventing abnormal accumulation of fatty acid in the liver.

## Author summary

Nonalcoholic fatty liver disease (NAFLD) is a disease associated with abnormal fat accumulation in the liver and other metabolic symptoms. Among its many social–behavioral and genetic causes, dysregulation of peroxisome proliferator-activated receptor γ (PPARγ) is an investigative focal point for therapeutic intervention. This lipid-sensing nuclear receptor plays a major role in promoting lipogenesis in adipose tissues, whereas its expression is low in the liver. We show here that in the absence of ubiquitin ligase (E3) Smurf1, PPARγ expression increases dramatically in the liver, causing fatty acid uptake and fat accumulation in hepatocytes. We also found that the low SMURF1 expression in human populations correlates with high body mass index (BMI) values. We demonstrate that Smurf1 catalyzes the lysine 63 (K63)-linked non-proteolytic modification of PPARγ that suppresses the transcriptional activity of PPARγ and breaks the positive feedback loop governing its own expression. Our data further indicate that treating this mouse model with a PPARγ antagonist, GW9662, completely reverses the fat accumulation in the liver.

## Introduction

Nonalcoholic fatty liver disease (NAFLD) is a chronic liver condition associated with obesity, non–insulin-dependent diabetes, and hyperglyceridemia [1]. Although presenting few clinical symptoms at early stages, a subset of patients with NAFLD will progress to nonalcoholic steatohepatitis (NASH) consisting of hepatic steatosis and inflammation, which can ultimately lead to cirrhosis and even liver cancer [2]. Myriad social–behavioral and genetic causes of NAFLD are now known, but the roles of peroxisome proliferator-activated receptors (PPARs) have emerged as crucial molecular underpinnings of these metabolic imbalances and targets of several investigational drugs [3–5]. A thorough understanding of regulatory mechanisms governing PPAR activities will undoubtedly aid in the development of much-needed treatments.

PPARs are nuclear hormone receptors that heterodimerize with retinoid X receptors to modulate metabolic transcriptional programs in response to nutritional inputs [6]. Of three PPARs encoded by distinct mammalian genes, PPARα, which is highly expressed in the liver, kidney, and muscle, directs expression of a network of genes that promote utilization of fat as an energy source. PPARγ, on the other hand, is normally expressed in adipose tissues, where it activates target genes involved in fatty acid uptake, transport, and lipogenesis to promote lipid storage. In the liver, PPARγ expression is normally low but becomes drastically induced as hepatic steatosis develops [7]. Reports in the literature have shown that overexpression of PPARγ promotes the accumulation of lipid droplets in the liver, whereas hepatic disruption of PPARγ improves the fatty liver condition in leptin-deficient obese mice or mice that were fed on a high-fat diet (HFD) [8, 9]. In adipose tissues, ligand binding was reported to induce degradation of PPARγ via the ubiquitin-proteasome system, whereas small ubiquitin-like modifier (SUMO)ylation of PPARγ was shown to repress its transcriptional activity [10]. However, how steatogenic activities of PPARγ are regulated in the liver remains to be determined.

Smad ubiquitin regulatory factor 1 (Smurf1) and its close relative, Smurf2, are members of homologous to E6-AP carboxyl terminus (HECT) domain–containing ubiquitin ligases (E3s), which were initially identified as negative regulators of transforming growth factor-β (TGF-β) and bone morphogenetic protein (BMP) signaling pathways [11–14]. Subsequent studies broadened the repertoire of Smurf substrates and extended their function to cell differentiation, polarity, and DNA repair [15–18]. During our ongoing quest for physiological functions of Smurfs, we found abnormal accumulation of lipid droplets in the livers of 9–12-month-old Smurf1 knockout (KO) mice and other signs that phenocopy NAFLD in human patients. Here, we report that Smurf1 induces non-proteolytic ubiquitination of PPARγ and inhibits PPARγ transcriptional activity in hepatocytes, thereby acting as a critical safeguard against the development of hepatic steatosis.

## Results

### NAFLD-like phenotypes associated with the loss of Smurf1

**Fig 1:**
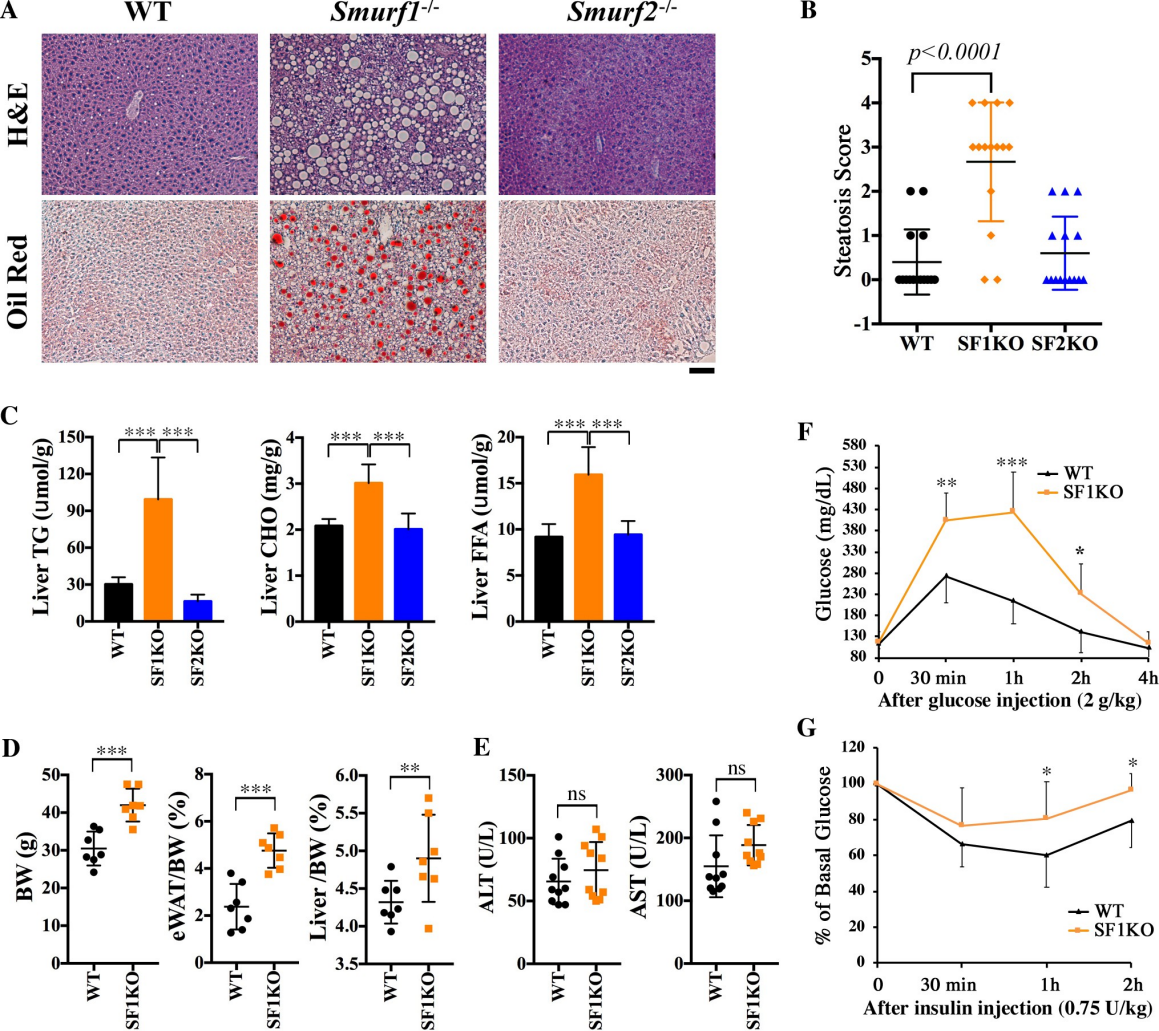
Smurf1KO mice in the mixed BL background developed hepatic steatosis. **(A)** HE and Oil Red-O staining of liver sections of WT, SF1KO, and SF2KO mice from the BL background at the age of 9–12 months. Bar = 40 μm. **(B)** Histological scores of steatosis of mice in (A). For each group, *n* = 15 (7 males and 8 females). Scores: 0, no steatosis; 1, minimal; 2, mild; 3, moderate; 4, severe. **(C)** Liver TG, CHO, and FFA content of the above male mice (*n* = 7 per group). **(D)** BW, eWAT/BW ratio, and Liver/BW ratio of the above male mice (*n* = 7 per group). **(E)** Liver ALT and AST activities of the above mice (*n* = 10; 5 males, 5 females per group). **(F)** Glucose and **(G)** insulin tolerance tests in male mice at the age of 9–12 months (*n* = 8 per group). All data are presented as mean ± SD; statistical significance of difference is indicated as *p < 0.05, **p < 0.01, ***p < 0.001. Original raw data can be found in S1 Data. ALT, alanine transaminase; AST, aspartate transaminase; BL, mixed black Swiss × 129/SvEv background; BW, body weight; CHO, total cholesterol; eWAT/BW, epididymal WAT weight to body weight; FFA, free fatty acid; HE, hematoxylin–eosin; KO, knockout; Liver/BW, liver weight to body weight; ns, not significant; Smurf, Smad ubiquitin regulatory factor; SF1KO, Smurf1 KO; SF2KO, Smurf2 KO; TG, triglyceride; WAT, white adipose tissue; WT, wild-type.

We previously reported an increased bone density phenotype in aged Smurf1KO mice that were commonly observed under mixed black Swiss × 129/SvEv (BL) and C57BL/6N (B6) genetic background [18]. Further analysis revealed a conspicuous accumulation of lipid droplets in the livers of aged Smurf1KO mice that was unique to the BL background (S1 Table). The liver sections of these mice were characterized by large, clear, sharp-bordered cytoplasmic vacuoles upon hematoxylin–eosin (HE) staining (Fig 1A). The bright red staining of frozen sections by Oil Red-O confirmed the high fat and neutral lipid content therein (Fig 1A). This phenotype was observed in 12 out of 15 male and female mice examined beyond 9 months of age, implying a 75% penetrance. Microscopic quantification of HE-stained sections reaffirmed the statistically significant increase of steatosis in the livers of Smurf1KO mice compared with that of the wild-type (WT) controls (Fig 1B). Surprisingly, this steatosis phenotype was not observed in the livers of Smurf2KO mice (Fig 1A and 1B), suggesting that it is specifically associated with disruption of the Smurf1 function. To determine which lipid fractions were increased, we carried out colorimetric assays in liver lipid extracts prepared from Smurf1KO mice at 9–12 months of age, and the results showed that the level of triglycerides (TG) increased more than 3-fold compared with that of WT or Smurf2KO mice (Fig 1C). Moreover, the levels of total cholesterol (CHO) and free fatty acids (FFAs) were also increased significantly in Smurf1KO livers (Fig 1C). Compared with WT mice, Smurf1KO mice were approximately 30% heavier in body weight, bore more white adipose tissue (WAT), and had a higher liver to body weight ratio (Fig 1D). Nevertheless, despite exhibiting ostensible steatosis, the mutant livers appeared to function normally, as indicated by aspartate transaminase (AST) and alanine transaminase (ALT) activity measurements (Fig 1E). Because the manifestation of hepatic steatosis is usually accompanied by a constellation of adverse alterations in glucose metabolism, we conducted glucose tolerance and insulin resistance tests. At the fasting state, there was not much difference in plasma glucose levels between aged (9–12 months old) WT and Smurf1KO mice that had developed steatosis; however, following intraperitoneal injection of glucose, the blood glucose level of the mutant mice showed a more dramatic flash increase of the blood glucose level within 30 minutes of injection and more than 100% accumulative gain in the area under the curve (AUC) (Fig 1F). On the other hand, after an initial dip following the insulin injection, the blood glucose level in aged mutant mice recovered more rapidly and to a higher extent than that in WT controls (Fig 1G). The AUC of the insulin resistance test of aged Smurf1KO mice was 13.5% more compared with that of WT mice. Because young Smurf1KO mice (at 4–5 months of age) that had yet to develop steatosis scored no difference from their WT counterparts in both the tests (S1 Fig), the systemic change in glucose metabolism observed in aged mutant mice was most likely associated with the steatosis. Taken together, the phenotypes of hepatic steatosis, obesesity, glucose intolerance, and insulin resistance make these aged Smurf1KO mice a good mouse model of NAFLD.

### Ablation of Smurf1 exacerbates HFD-induced hepatic steatosis

**Fig 2:**
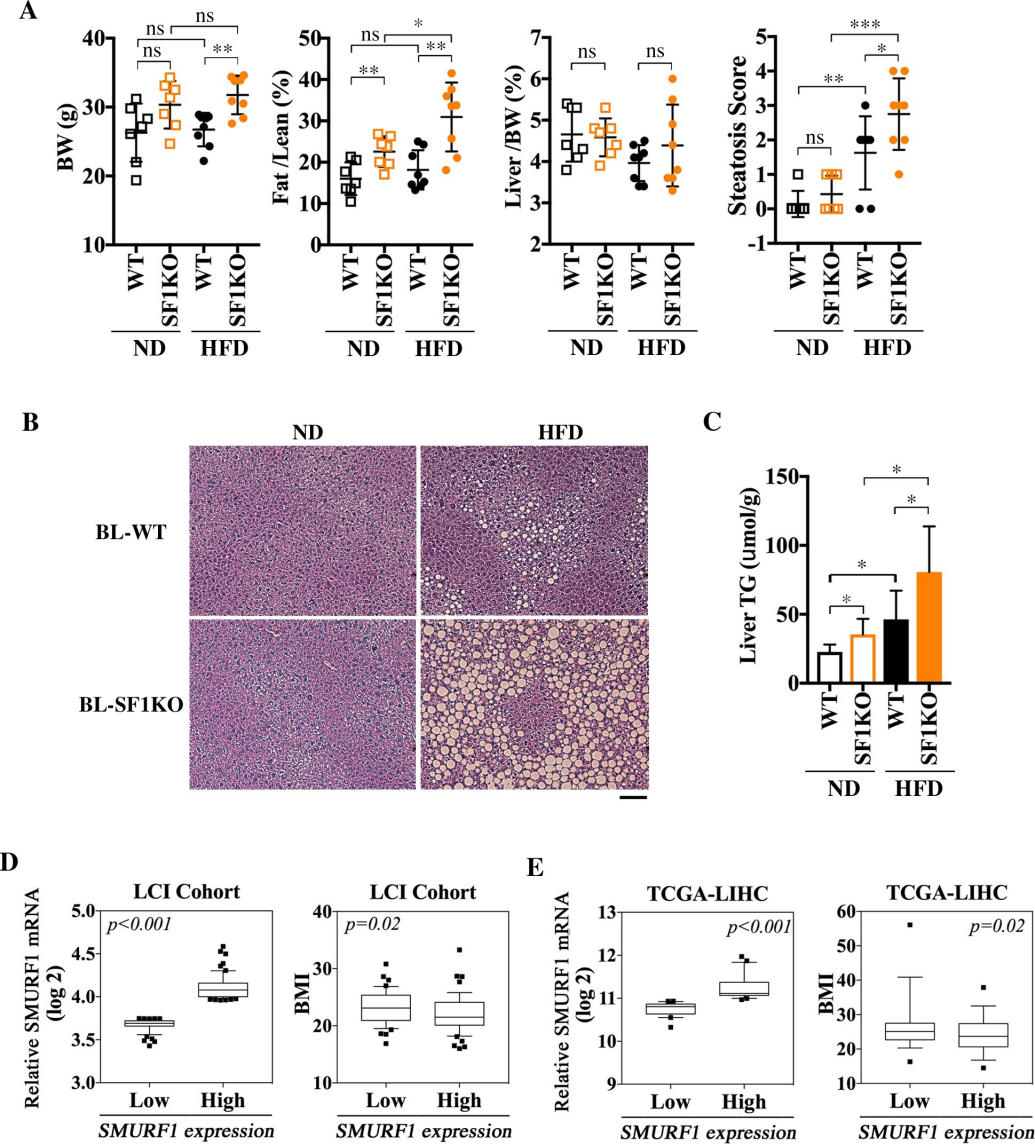
Inverse correlation of low Smurf1 expression to high fat accumulation. **(A–C) Loss of Smurf1 exacerbates HFD-induced steatosis in mice.** (A) BW, Fat/Lean ratio, Liver/BW ratio, and histological scores of steatosis in male WT and SF1KO mice from BL background reared on either a ND (*n* = 7 per group) or HFD (*n* = 8 per group), beginning at 10–12 weeks of age for 8 weeks. Liver sections were scored as in Fig 1B. **(B)** HE staining of representative liver sections of the above BL mice at the end of diet treatment. Bar = 100 μm. **(C)** Liver TG levels of BL mice from (A). Data are presented as mean ± SD; statistical significance of differences is indicated as *p < 0.05, **p < 0.01, ***p < 0.001. Original raw data can be found in S1 Data. **(D–E)** Low SMURF1 expression in human livers is inversely correlated to high BMI values. (D) Human liver tissues from the LCI cohort with low levels of SMURF1 expression show a significantly higher BMI than those with high SMURF1 expression. Non-tumor liver samples from the LCI cohort were grouped into SMURF1 High (top 25%, *n* = 61) and SMURF1 Low (bottom 25%, *n* = 59) groups. **(E)** Inverse correlation between SMURF1 low and BMI high was also observed in non-tumor liver samples from the TCGA-LIHC cohort. Non-tumor liver samples (*n* = 37) from the TCGA-LIHC cohort were grouped into SMURF1 High (above the median, *n* = 18) and SMURF1 Low (below the median, *n* = 19) groups. Data are presented using box and whisker plot; centerline represents the median, whiskers represent 10th–90th percentile. Nonparametic *t* tests between the two groups were performed; *p*-values are indicated in the graph. BL, mixed black Swiss × 129/SvEv background; BMI, body mass index; BW, body weight; Fat/Lean, fat mass to lean mass; HE, hematoxylin–eosin; HFD, high-fat diet; KO, knockout; LCI, Liver Cancer Institute; Liver/BW, liver weight to body weight; ND, normal diet; ns, not significant; SF1KO, Smurf1 KO; Smurf, Smad ubiquitin regulatory factor; TG, triglyceride; TCGA-LIHC, the cancer genome atlas-liver hepatocellular carcinoma; WT, wild-type.

In rodents, difference in genetic background has a well-known influence on the susceptibility to obesity and hepatic steatosis [19–21]. Although the spontaneous steatosis hereto described was only observed at old age, young Smurf1KO mice of the BL background were grossly normal except for a higher body fat content compared with their age- and background-matched WT counterparts and showed no sign of steatosis (Fig 2A and 2B) when fed on normal diet (ND). Mice of this strain background are notoriously known for their resistance to HFD-induced obesity, as evident by the lack of apparent gain in body weight and ratio of fat-to-lean mass in young WT mice that were put on a HFD feeding regimen beginning at 10–12 weeks of age and continuing for 8 consecutive weeks (Fig 2A, S2 Table). In contrast, HFD feeding significantly increased fat content in the Smurf1KO mice (Fig 2A, S2 Table). Despite a lack of significant weight gain, HFD feeding did cause mild steatosis (Fig 2A and 2B), as well as an increase in liver TG content in WT mice (Fig 2C); however, these changes were all dramatically exacerbated in BL-Smurf1KO mice (Fig 2A–2C).

As alluded earlier, Smurf1KO mice of the B6 background did not show accumulation of lipid droplets in the liver (S1 Table), and they were not overweight or overtly obese either (S2A Fig). To ascertain that the steatosis pheneotype was not a mere coincidence unique to the BL background, we carried out the HFD feeding study on WT and Smurf1KO mice of the B6 background with the same regimen as for the young BL mice. In contrast to BL mice, B6 mice gained body weight and fat content on HFD as expected, regardless of the presence of Smurf1 gene (S2A Fig, S2 Table). However, the B6-Smurf1KO mice on HFD became ostensibly more obese (S2A Fig) and showed more severe lipid droplet accumulation in the liver compared to WT mice of the same background (S2A and S2B Fig). In addition, the increase in the liver TG content was also more pronounced (S2C Fig). Thus, the steatosis associated with Smurf1 loss is likely the result of an overall gain in body fat content in both strain backgrounds, suggesting that Smurf1 may have a systemic role in regulating lipid metabolism.

### Human SMURF1 expression inversely correlates with body mass index

To address if what we learned from the Smurf1KO mice is applicable to human populations, we took the advantage of the non-tumor liver tissue data sets compiled from a cohort of 247 Chinese liver cancer patients from the Liver Cancer Institute (LCI) [22]. According to the SMURF1 mRNA expression levels retrieved from the gene expression profile (GEO: GSE14520), we separated non-tumor liver tissue samples into the high SMURF1 expression (top 25%) group (*n* = 61) and the low SMURF1 expression (bottom 25%) group (*n* = 59) (Fig 2D, left panel). We then graphed the body mass index (BMI) of these 120 patients against these two groups of SMURF1 expression, and found that patients with the low SMURF1 expression have a statistically significant higher BMI (Fig 2D, right panel). It is worth noting that the average BMI of the Asian population is lower than that in the United States and European countries, and an Asian with BMI > 27.5 is considered obese [23, 24]. This inverse correlation was further corroborated with non-tumor liver tissue data sets from the cancer genome atlas-liver hepatocellular carcinoma (TCGA-LIHC) (Fig 2E). Because there are only 37 cases of non-tumor liver samples that have linked BMI values in the TCGA data set, the median SMURF1 expression level was used as the cutoff to plot BMI values (Fig 2E). Because the BMI is widely used in clinics as a surrogate prognostic indicator for fatty liver [25, 26], these results suggest that low Smurf1 expression appears to be associated with high fat accumulation in humans, as well.

### Loss of Smurf1 activates the PPARγ pathway

**Fig 3:**
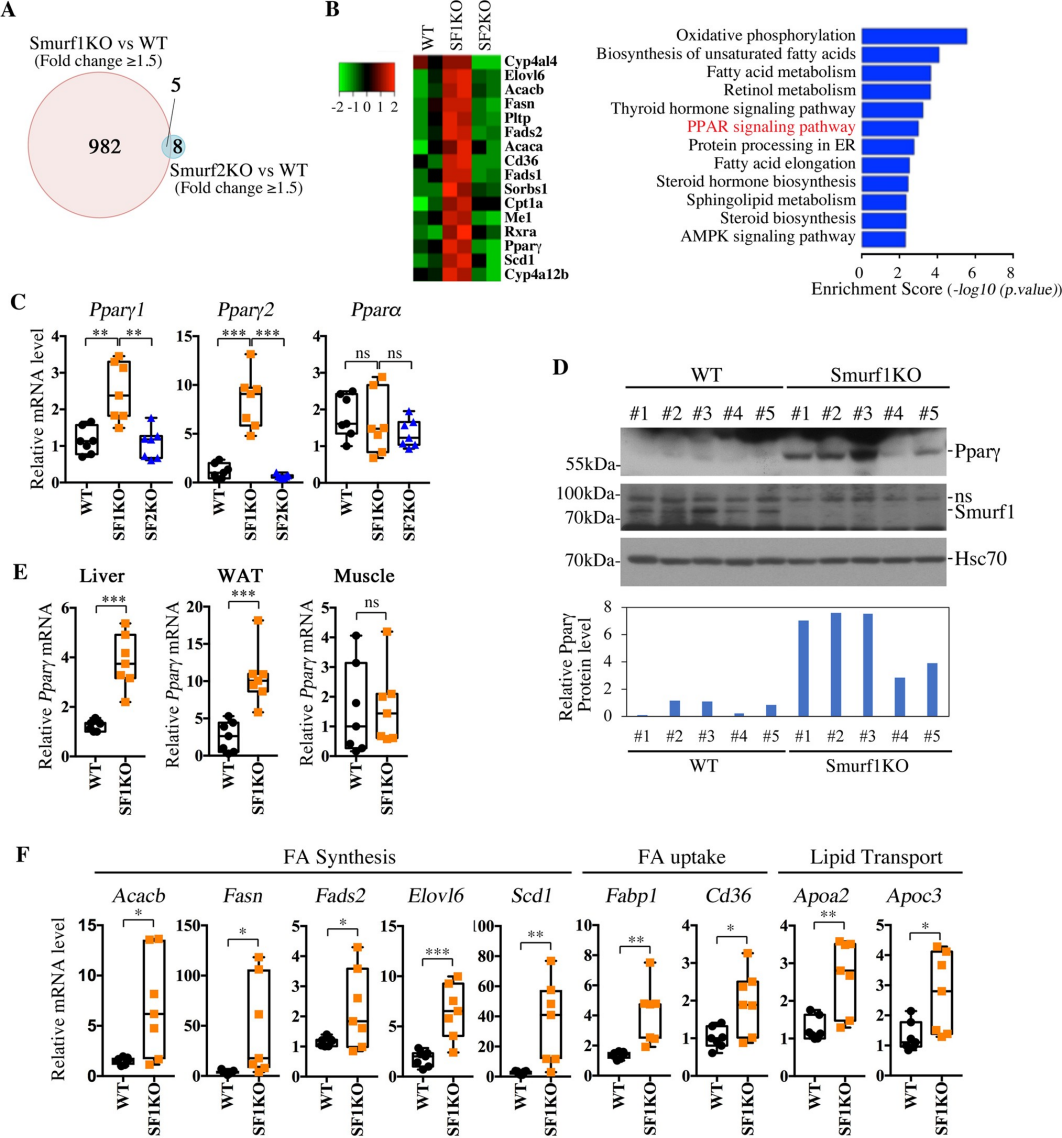
Up-regulation of PPARγ and other relevant lipid metabolic pathways associated with Smurf1 loss. **(A)** Venn diagram of genes that were differentially expressed (fold > 1.5, FDR < 0.1) in Smurf1KO and Smurf2KO livers from the BL background relative to their WT counterparts at 11 months of age. For a detailed list, see S3 Table. **(B)** Heat map of a group of lipid metabolism–related genes that were differentially expressed in Smurf1KO livers and the KEGG pathway analysis of differentially expressed genes from BL-Smurf1KO livers. Enrichment score (*p*-value (−log10)) is indicated on the x-axis. **(C)** qRT-PCR analyses of *Ppar*γ*1*, *Ppar*γ*2*, and *Ppar*α in livers from BL-WT, Smurf1KO, and Smurf2KO mice (*n* = 7 per group). **(D)** Western blot showing the increase of the PPARγ protein in livers of BL-Smurf1KO mice. Quantitation of PPARγ expression against loading control Hsc70 is shown at the bottom. **(E)** qRT-PCR analyses showing the increase of total *Ppar*γ mRNA in livers and epididymal WAT but not skeletal muscle of BL-Smurf1KO mice, *n* = 7 per group. **(F)** qRT-PCR analyses showing up-regulation of a group of PPARγ target genes in livers of BL-Smurf1KO mice (*n* = 7 per group). Data from qRT-PCR are presented using box and whisker plot showing all points; centerline represents the median. All mice were analyzed at 9–11 months of age, and statistical significance of difference between WT and Smurf1KO is indicated as *p < 0.05, **p < 0.01, and ***p < 0.001. Original raw data can be found in S1 Data. BL, mixed black Swiss × 129/SvEv background; FDR, false discover rate; Hsc70, heat shock 70 kDa protein 8; KEGG, Kyoto Encyclopedia of Genes and Genomes; KO, knockout; ns, nonspecific band; PPAR, peroxisome proliferator-activated receptor; qRT-PCR, quantitative real-time PCR; SF1KO, Smurf1 KO; SF2KO, Smurf2 KO; Smurf, Smad ubiquitin regulatory factor; WAT, white adipose tissue; WT, wild-type.

To investigate the underlying causes of steatosis associated with Smurf1 loss, we compared hepatic gene expression profiles of 11-month-old Smurf1KO, Smurf2KO, and their respective matching WT mice from the BL background, and selected genes that showed either increased or decreased expression by a cutoff of 1.5-fold (false discover rate [FDR] <0.1). The results showed that 987 genes in the Smurf1KO livers were differentially expressed over their WT controls, whereas only 13 genes were differentially expressed in the Smurf2KO livers (Fig 3A, left panel, and S3 Table). This result is in line with the notion that Smurf1 plays a more prominent role in the liver than Smurf2. Many genes that are involved in the lipid metabolism were up-regulated in Smurf1KO livers, and the Kyoto Encyclopedia of Genes and Genomes (KEGG) pathway enrichment analysis of differentially expressed genes between Smurf1KO and WT livers revealed a number of metabolically relevant pathways (Fig 3B). We were intrigued by the enrichment of the PPAR signaling pathway that has known strong effects on steatosis [4]. Of the three PPAR genes, *Ppar*γ encodes two protein isoforms, PPARγ1 and PPARγ2, whose mRNAs are transcribed from two separate promoters [27, 28]. Quantitative real-time PCR (qRT-PCR) analyses showed severalfold increases of both *Pparγ* isoforms in the livers of aged Smurf1KO but not Smurf2KO mice (Fig 3C). Interestingly, the expression of *Ppar*α was not altered in the liver of any mouse examined (Fig 3C). In young BL mice (10–12 weeks of age) that had yet to develop steatosis, loss of Smurf1 increased the expression of total *Ppar*γ (about 1.57-fold) when the mice were fed on ND (S3A Fig), suggesting that Smurf1 has a direct causal effect on *Ppar*γ expression. HFD feeding further exacerbated the difference of *Ppar*γ expression to 3.42-fold between WT and Smurf1KO livers (S3A Fig). On the other hand, no difference was observed in TNFα and F4/80 expression (S3B Fig), two genes involved in inflammatory response, which is consistent with the absence of any inflammation in Smurf1KO mice (S1 Table and S1 Data). Western blot analyses confirmed the corresponding up-regulation of the PPARγ protein in the livers of aged Smurf1KO mice (Fig 3D). According to data from The Human Protein Atlas (https://www.proteinatlas.org/ENSG00000198742-SMURF1/tissue), Smurf1 protein is highly expressed in visceral organs, but its expression levels in muscle and adipose tissues are extremely low or moderate, respectively. This likely accounts for the dramatic increase of PPARγ in the Smurf1KO livers, where Smurf1 function is expected to be robust. Consistent with tissue distribution of Smurf1 expression, qRT-PCR revealed that total *Ppar*γ expression increased dramatically in the liver and WAT but did not change in the muscle of Smurf1KO mice (Fig 3E). Finally, loss of Smurf1 cast a profound impact on the hepatic expression of PPARγ transcriptional target genes that are involved in fatty acid synthesis, uptake, and transport (Fig 3F), thus lending further support to the activation of PPARγ and its signaling pathway in aged Smurf1KO mice.

### Smurf1 regulates fatty acid uptake and lipid synthesis through PPARγ

**Fig 4:**
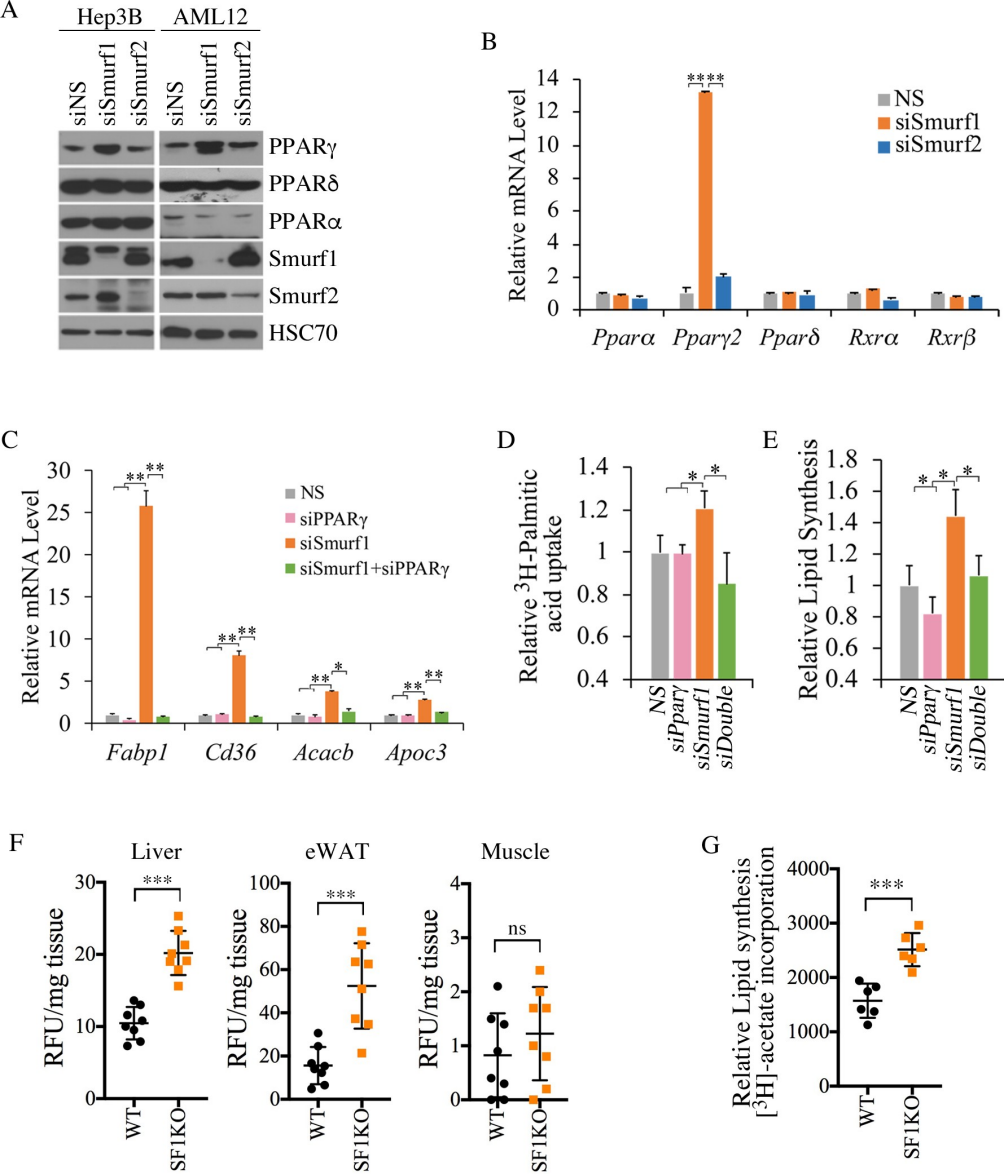
Smurf1 regulates fatty acid uptake and lipid synthesis through PPARγ. **(A)** Western blots showing that knockdown of Smurf1 but not Smurf2 in Hep3B and AML12 cells increased PPARγ protein level. **(B)** qRT-PCR analyses showing that knockdown of Smurf1 but not Smurf2 increased *Ppar*γ mRNA level in AML12 cells. **(C)** qRT-PCR analyses showing that knockdown of Smurf1 in AML12 cells increased expression of *Fabp1*, *Cd36*, *Acacb*, and *Apoc3* in a PPARγ-dependent manner. **(D)** Fatty acid uptake in AML12 cells as measured by ^3^H-palmitate incorporation (*n* = 3). (E) Lipid synthesis in AML12 cells as measured by incorporation of ^3^H-acetate into lipid (*n* = 3). **(F)** In vivo fatty acid uptake after intraperitoneal injection of BODIPY-FL-C16. The BODIPY-FL-C16 accumulation in the liver, epididymal WAT, and skeletal muscle was normalized to tissue weight (*n* = 8 per group). **(G)** Lipogenesis in primary hepatocytes as measured by the incorporation of ^3^H-acetate into lipid (*n* = 6 per group). Data are presented as mean ± SD; statistical significance of difference is indicated as *p < 0.05, **p < 0.01, ***p < 0.001. Original raw data can be found in S1 Data. BODIPY-FL-C16, 4,4-Difluoro-5,7-Dimethyl-4-Bora-3a,4a-Diaza-*s*-Indacene-3-Hexadecanoic Acid; eWAT, epididymal WAT; HSC70, heat shock cognate 71 kDa protein; NS, non-silencing control; PPAR, peroxisome proliferator-activated receptor; qRT-PCR, quantitative real-time PCR; RFU, relative fluorescence units; Rxr, retinoid x receptor; SF1KO, Smurf1 KO; siNS, non-silencing control siRNA; Smurf, Smad ubiquitin regulatory factor; WAT, white adipose tissue; WT, wild-type.

PPARγ is a strong lipogenic factor essential for steatosis [7]. Although our qRT-PCR analysis alluded that loss of Smurf1 has a direct causal effect on PPARγ1 up-regulation, further evidence is needed to confirm this finding. Toward this end, we silenced Smurf1 using short interfering RNA (siRNA)s in human hepatocarcinoma Hep3B cells and mouse normal hepatocyte AML12 cells. Relative to the effect by non-silencing control siRNA (siNS), knockdown by siSmurf1 significantly increased the level of PPARγ but not PPARα or PPARδ in both cell lines (Fig 4A). As expected, siSmurf2 had little effect in either of these two cell lines (Fig 4A). Because *Ppar*γ is a direct transcriptional target of itself in a positive feedback loop [29], siRNA-mediated silencing of Smurf1 drastically increased the expression level of *Ppar*γ, but not other paralogous *Ppars* or their transcriptional regulatory partners retinoid x receptor (*Rxr*)α and *Rxr*β (Fig 4B). In adipose tissues, transcription of *Ppar*γ genes is under the control of CCAAT enhancer binding protein (CEBP)α/β [30, 31]; however, we were unable to detect any increase of either *Cebp*α or *Cebp*β mRNA by qRT-PCR (S4A Fig), suggesting that the regulation of PPARγ by Smurf1 is by way of a C/EBPα/β-independent mechanism. In line with the low expression of PPARγ in AML12 cells, introducing siPPARγ showed little effect on the expression of PPARγ transcriptional target genes, *Fabp1*, *Cd36*, *Acacb*, and *Apoc3*, but siSmurf1 significantly increased the expression of these genes (Fig 4C, and S4B Fig). Furthermore, introducing siPPARγ completely blocked the enhancing effect of siSmurf1 (Fig 4C), thus confirming the direct causal relationship between Smurf1 and PPARγ. The fact that up-regulation caused by siSmurf1was particularly pronounced in *Fabp1* and *Cd36*, two genes that are essential for fatty acid uptake [32, 33], suggested a strong connection between Smurf1 and fatty acid uptake. Indeed, using ^3^H-labelled palmitic acid as a tracer, we observed a 20% increase in fatty acid uptake by AML12 cells upon Smurf1 depletion (Fig 4D). We also measured lipid synthesis in AML12 cells by measuring the incorporation of ^3^H-labelled acetate into lipids and found it was increased by siSmurf1 as well (Fig 4E). Once again, these two effects of Smurf1 loss were specifically mediated through PPARγ as they were reversed by siPPARγ (Fig 4D and 4E).

To further show if Smurf1 actually regulates lipid metablism in vivo, we injected fluorescent 4,4-Difluoro-5,7-Dimethyl-4-Bora-3a,4a-Diaza-*s*-Indacene-3-Hexadecanoic Acid (BODIPY-FL-C16) into the peritoneal cavities of WT and Smurf1KO mice and found that the fatty acid uptake was greatly enhanced in the liver and WAT tissues but not the muscles of Smurf1KO mice compared with that of WT mice (Fig 4F). We also repeated the ^3^H-labelled acetate incorporation experiment in primary hepatocytes isolated from WT and Smurf1KO mice and confirmed the enhancement effect of Smurf1 ablation on lipid synthesis (Fig 4G).

The increased body fat content in aged BL-Smurf1KO mice and HFD-fed young Smurf1KO mice from both background suggests that Smurf1 may also regulate adipogenesis. To determine if this was the case, we took advantage of an in vitro adipogenic differentiation system using 3T3-L1 pre-adipocytes. Following a 6-day differentiation protocol, both PPARγ1 and PPARγ2 as well as their target Cd36 were all induced, as shown by western blot analysis, and the induction was greatly enhanced by siSmurf1 but reversed by the double transfection of siSmurf1 and siPPARγ (S5A Fig). In keeping with the western blot analysis results, Oil Red-O staining of these differentiated 3T3-L1 cells was also enhanced by siSmurf1 and reversed by siSmurf1 and siPPARγ double transfection (S5B Fig). Finally, expression of a cohort of adipogenic target genes of PPARγ also followed the same pattern as influenced by siSmurf1 and siPPARγ (S5C Fig). Taken together, these data indicate that Smurf1 has an intrinsic role in controlling adipogenesis and lipid metabolism through PPARγ.

### PPARγ is a direct substrate of Smurf1-mediated non-proteolytic ubiquitination

**Fig 5:**
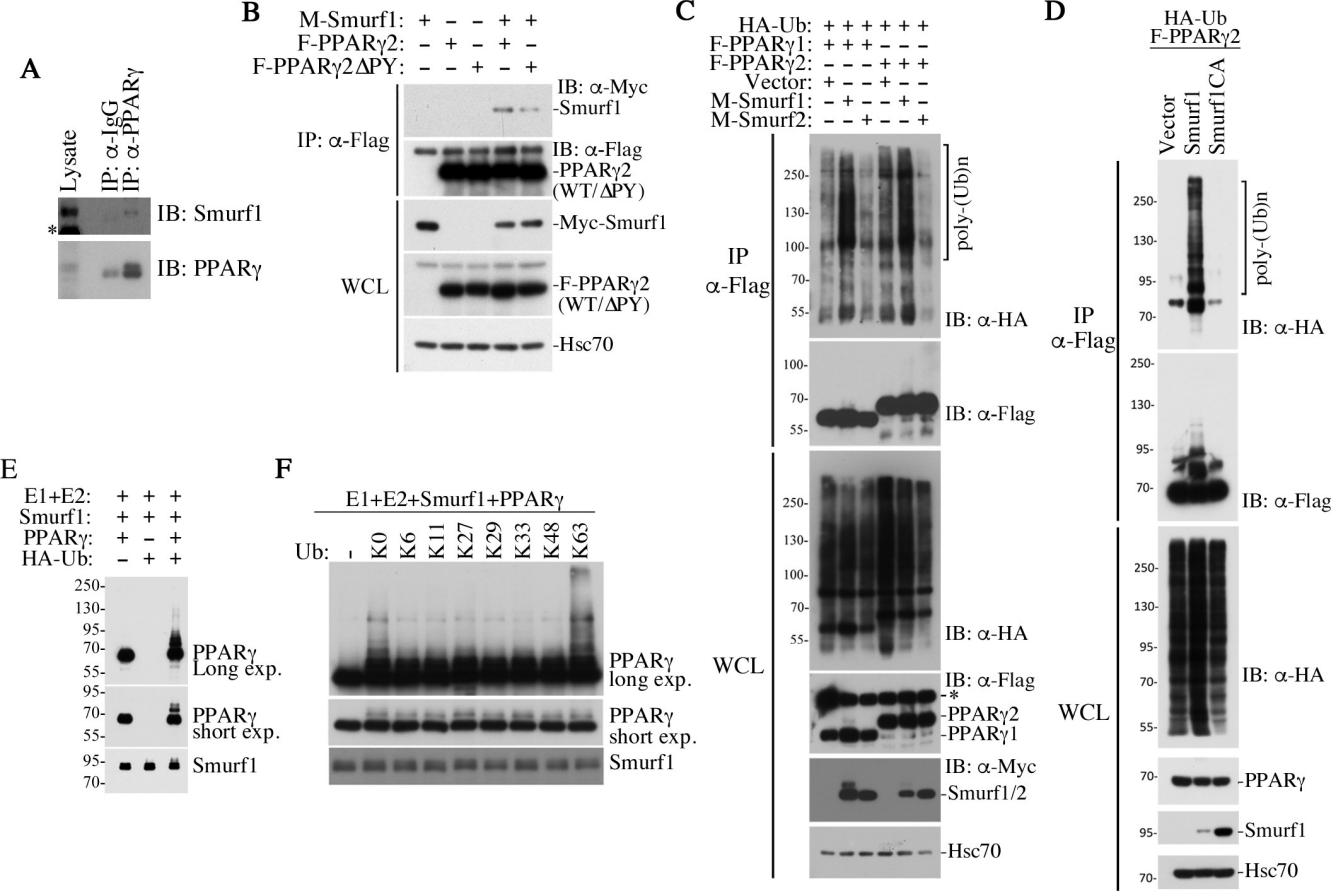
Smurf1 catalyzes non-proteolytic ubiquitination of PPARγ. **(A)** Co-immunoprecipitation showing interaction between endogenous Smurf1 and PPARγ in AML12 cells. *nonspecific band. **(B)** PY motif in PPARγ contributes to the interaction between Smurf1 and PPARγ. Myc-tagged Smurf1, Flag-tagged PPARγ2, and its ΔPY mutant were transfected into AML12 cells as indicated. WCL were immunoprecipitated with Flag-M2 beads and followed by western blot analyses. **(C)** Smurf1 but not Smurf2 promotes polyubiquitination of PPARγ1 and PPARγ2. Flag-PPARγ were immunoprecipitated from transfected AML12 and resolved by SDS-PAGE. Western blot analyses were carried out to detect HA-ubiquitin (top) and Flag-PPARγ1 or -γ2 (second panel) in the precipitates. The levels of total HA-Ub, Flag-PPARγ, Myc-Smurfs, and endogenous Hsc70 (loading control) in the WCL were also analyzed and are shown in the bottom panels. *nonspecific band. **(D)** E3 ligase activity of Smurf1 is required for Smurf1-mediated polyubiquitination of PPARγ. Flag-PPARγ2 and WT Myc-Smurf1 and its mutant Myc-Smurf1(CA) were transfected into the Smurf1KO MEFs along with HA-Ub. Ubiquitination of PPARγ2 was analyzed by western blot after Flag-M2 immunoprecipitation, as in C. **(E)** In vitro ubiquitination assay using recombinant proteins showing that PPARγ is a direct substrate of Smurf1-mediated polyubiquitination. **(F)** Smurf1 induces K63-linked polyubiquitination of PPARγ. Purified ubiquitin with no lysine residue (K0) or with single lysine residue at indicated position was used in the in vitro ubiquitination assay. E3, ubiquitin ligase; HA, human influenza hemagglutinin; Hsc70, heat shock cognate 71 kDa protein; IB, immunoblot; IP, immunoprecipitation; K, lysine; KO, knockout; MEF, mouse embryonic fibroblast; PPAR, peroxisome proliferator-activated receptor; PY, PPxY; SDS-PAGE, sodium dodecyl sulfate-polyacrylamide gel electrophoresis; Smurf, Smad ubiquitin regulatory factor; Ub, ubiquitin; WCL, whole cell lysate; WT, wild-type.

The WW domains of HECT E3 ligases recognize a PPxY (PY) motif that is present in the primary sequence of many of their targets [34]. There is one such sequence motif in both human and mouse PPARγ but not in PPARα, which might potentially account for the lack of an effect on this closely related protein by the loss of Smurf1 (Fig 3C). By co-immunoprecipitation experiments, we found that endogenous Smurf1 interacted specifically with PPARγ in the AML12 cells (Fig 5A), and the PY motif of PPARγ contributed to the interaction, because removing it considerably weakened the interaction between Myc-tagged Smurf1 and FLAG-tagged PPARγ, as assayed in transiently transfected AML12 cells (Fig 5B). Also in AML12 cells, Smurf1 but not Smurf2 showed the propensity to ubiquitinate both PPARγ1 and PPARγ2 isoforms (Fig 5C). The substrate and enzyme relationship was further demonstrated in Smurf1KO mouse embryonic fibroblasts (MEFs), in which exogenous Smurf1 but not the catalytically inactive Smurf1 C699A (CA) mutant ubiquitinated PPARγ (Fig 5D), as well as in a reconstituted in vitro reaction with recombinant Smurf1 and PPARγ (Fig 5E). Finally, the ubiquitin chain of the modified PPARγ is likely of the K63 linkage, as only the ubiquitin mutant with a single lysine residue at the amino acid residue position 63 supported the polyubiquitination of PPARγ in the reconstituted in vitro reaction, whereas other single-lysine ubiquitin mutants with lysine at other positions did not (Fig 5F). In light of this result and the fact that co-expressing Smurf1 with PPARγ did not alter the stability of the latter (Fig 5B and 5C), we concluded that Smurf1 mediates a non-proteolytic ubiquitin modification of PPARγ.

### Non-proteolytic modification by Smurf1 suppresses PPARγ-mediated transcription

**Fig 6:**
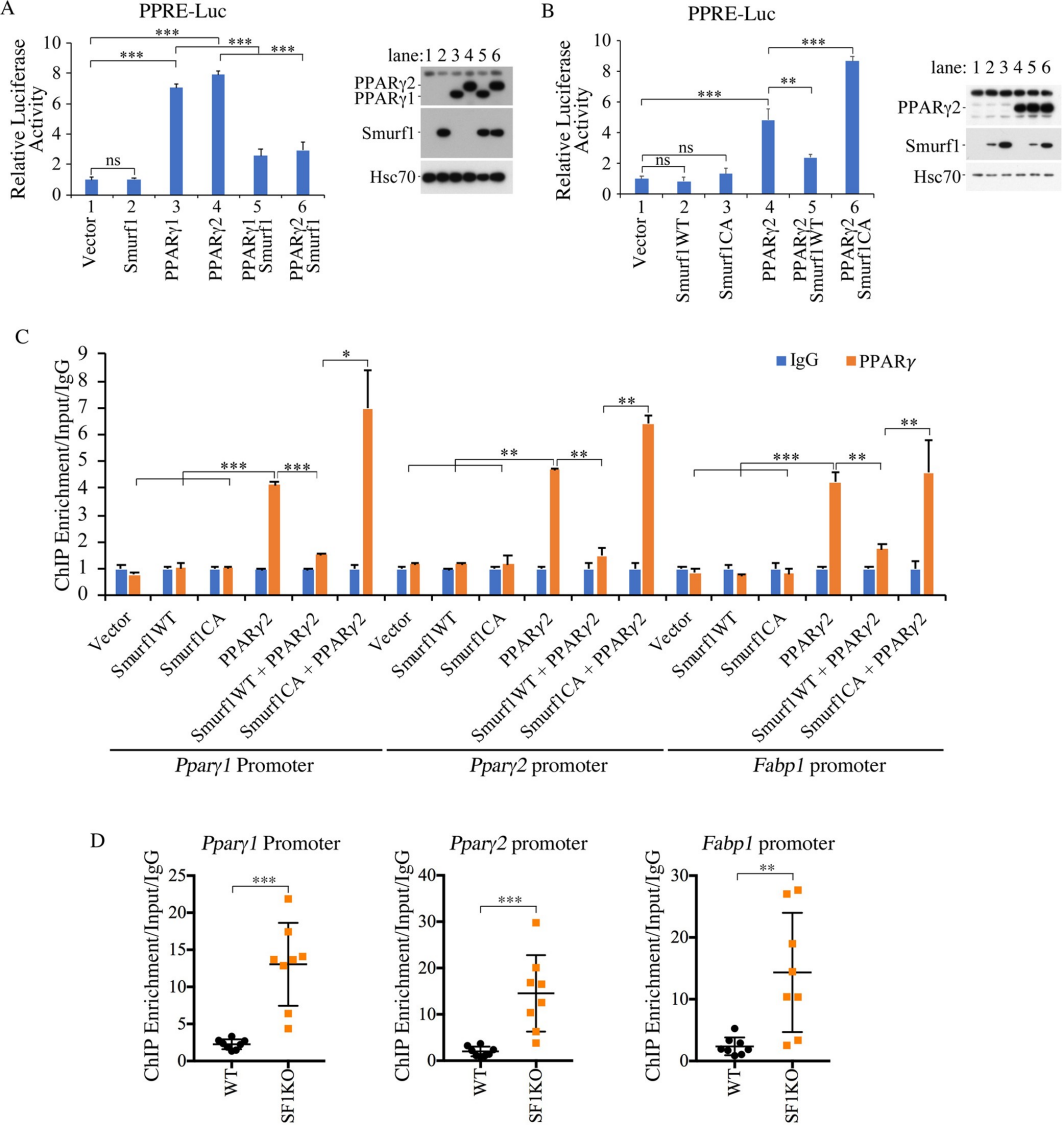
Smurf1 inhibits PPARγ transcriptional activity. **(A)** Smurf1 inhibits PPARγ-induced transcriptional activity in AML12 cells. Relative luciferease activities were measured 1 day after transfection. Data are presented as mean ± SD; statistical significance of differences is indicated by *p < 0.05, **p < 0.01, ***p < 0.001. Expression of transfected Smurf1 and PPARγ in these cells is shown at right. **(B)** E3 ligase activity of Smurf1 is required for its inhibition of PPARγ transcriptional activity. Luciferase activities were measured and showed as above. Expression of transfected Smurf1 and PPARγ in these cells are shown at right. **(C)** ChIP analyses of PPARγ binding to its own or Fabp1 promoter in AML12 cells after transfecting the plasmids as indicated. **(D)** ChIP analyses of PPARγ binding to its own or Fabp1 promoter in liver tissues from WT and Smurf1KO mice (*n* = 8 per group). Data are presented as mean ± SD; statistical significance of difference is indicated by *p < 0.05, **p < 0.01, ***p< 0.001. Original raw data can be found in S1 Data. ChIP, chromatin immunoprecipitation; E3, ubiquitin ligase; Hsc70, heat shock cognate 71 kDa protein; IgG, Immunoglobulin G; KO, knockout; PPAR, peroxisome proliferator-activated receptor; PPRE-Luc, PPAR response element-luciferase reporter; SF1KO, Smurf1 KO; Smurf, Smad ubiquitin regulatory factor; WT, wild-type.

PPARs recognize a consensus sequence of PPAR response element (PPRE) that consists of two AGGTCA-like sequences arranged in tandem with a single nucleotide spacer and is present in all PPAR target gene promoters [35, 36]. In AML12 cells, where PPARγ expression is very low, overexpressing Smurf1 had little effect on a luciferase reporter driven by PPRE, whereas both PPARγ1 and PPARγ2 significantly activated it; however, co-expressing Smurf1 with either PPARγ1 or PPARγ2 severely curtailed their transcriptional activity (Fig 6A). Because Smurf1 has no effect on PPARγ protein levels per se (Fig 6A, right panel), these results suggested that Smurf1 inhibits the transcriptional activity of PPARγ. The regulation by Smurf1 depends on its E3 ligase activity because a ligase-deficient mutant, Smurf1CA, could not reverse the activation of PPRE-luc by PPARγ (Fig 6B). Chromatin immunoprecipitation (ChIP) experiments on *Ppar*γ*1*, *Ppar*γ*2*, and *Fabp1* promoters indicated that the binding of PPARγ to these promoters was blocked when it was co-expressed with Smurf1 (Fig 6C). Once again, the E3 ligase activity of Smurf1 is required for its ability to block DNA binding of PPARγ (Fig 6C). ChIP experiments performed in liver extracts isolated from WT and Smurf1KO mice also revealed a much stronger binding of PPARγ to its own *Ppar*γ*1* and *Ppar*γ*2* promoters, as well as its target *Fabp1* promoter in the absence of Smurf1 (Fig 6D), thus lending further support to Smurf1 regulating transcriptional activity of PPARγ.

### PPARγ antagonist GW9662 protects Smurf1KO mice from hepatosteatosis

**Fig 7:**
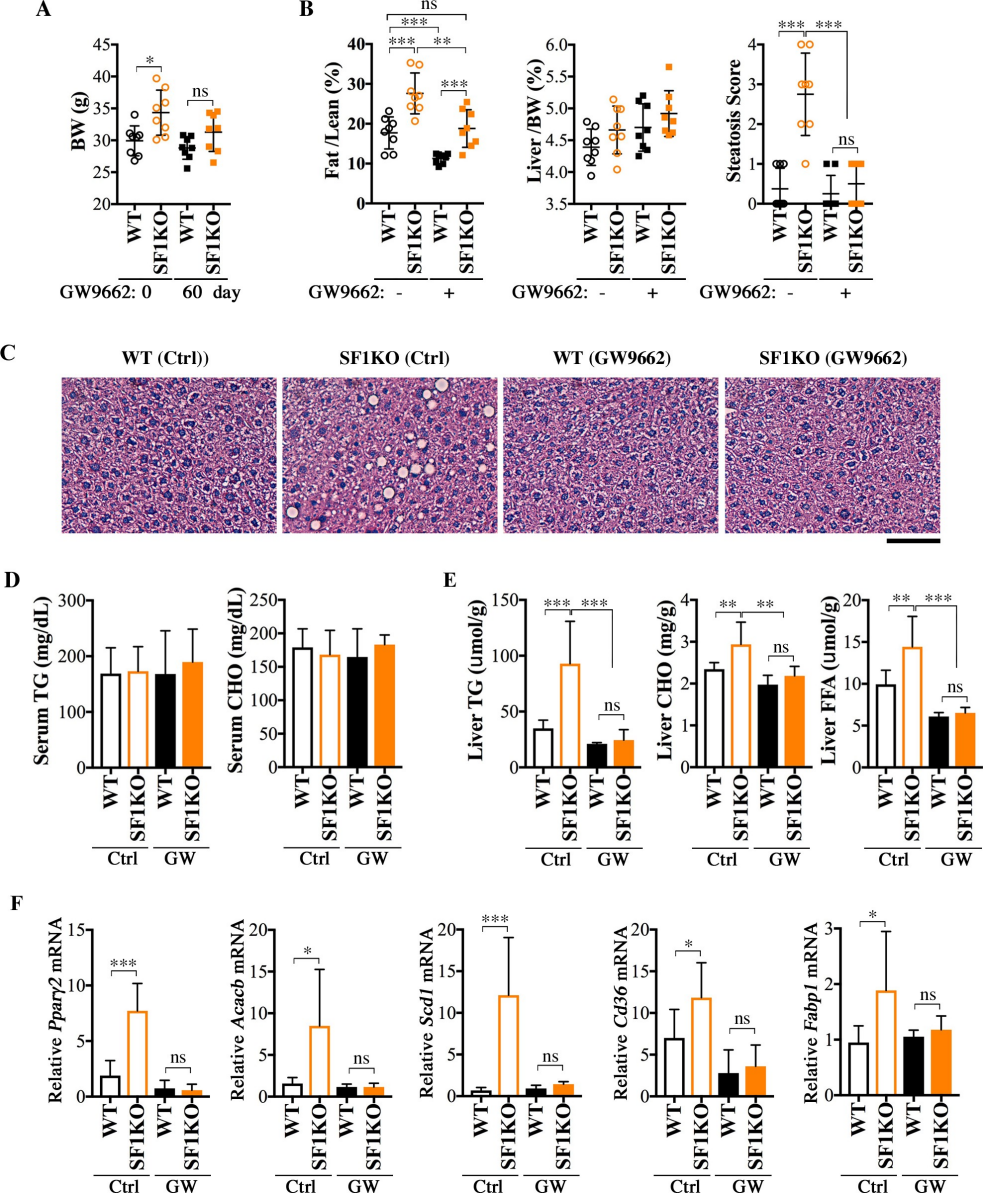
Treatment with PPAR antagonist GW9662 protects BL-Smurf1KO mice from steatosis. **(A)** BW of male mice from the BL background before (age 7–9 months) and after 60 days (age 9–11 months) of GW9662 treatment (*n* = 8 per group). **(B)** Fat/Lean and Liver/BW ratios and histological score of steatosis of the above mice after GW9662 treatment, compared with those of untreated control mice at the same age (*n* = 8 per group). **(C)** Representative HE-stained sections of the above mice. Bar = 100 μm. **(D)** Serum TG and CHO contents of the above mice, *n* = 8 per group. **(E)** Liver TG, CHO, and FFA contents of the above GW9662-treated mice. **(F)** qRT-PCR analysis of expression of *Ppar*γ*2* and relevant lipid metabolism genes in the livers of GW9662-treated mice. Data are presented as mean ± SD; statistical significance of difference is indicated by *p < 0.05, **p < 0.01, ***p < 0.001. Original raw data can be found in S1 Data. BL, mixed black Swiss × 129/SvEv background; BW, body weight; CHO, total cholesterol; Ctrl, control; Fat/Lean, fat mass to lean mass; FFA, free fatty acid; GW, GW9662; HE, hematoxylin–eosin; KO, knockout; Liver/BW, liver weight to BW; ns, not significant; PPAR, peroxisome proliferator-activated receptor; qRT-PCR, quantitative real-time PCR; SF1KO, Smurf1 KO; Smurf, Smad ubiquitin regulatory factor; TG, triglyceride; WT, wild-type.

To directly test if the increased PPARγ activity and expression are responsible for steatosis associated with Smurf1 loss, we treated a group of WT and Smurf1KO mice from the BL background with the PPARγ antagonist GW9662 [37]. The compound was administered by intraperitoneal injection starting at 7–9 months of age, and the treatment lasted for 2 months; in this time period, the steatosis was expected to fully develop in Smurf1KO mice. The GW9662 treatment decreased body weight of both WT and Smurf1KO mice (Fig 7A), but because the average beginning weight of Smurf1KO mice was higher, the reduction thereof was more dramatic than that of the WT controls (about 10% reduction versus about 5%). The body fat mass content in Smurf1KO mice was also significantly lowered, to an extent that was comparable to that of the untreated WT mice (Fig 7B). Commensurate to the systemic reduction in obesity, the lipid droplets were essentially cleared from Smurf1KO livers by GW9662 (Fig 7B and 7C). Although the GW9662 treatment caused no significant change in the serum TG and CHO levels (Fig 7D), hepatic contents of TG, CHO, and FFA were all reduced to normal levels (Fig 7E) and so was hepatic expression of *Ppar*γ*2*, as well as several PPARγ target genes (Fig 7F). These results unequivocally demonstrated that the elevated PPARγ activity and expression account for the NAFLD phenotypes observed in Smurf1KO mice.

## Discussion

PPARγ is a nuclear hormone receptor with principle functions of increasing insulin sensitivity and promoting lipid storage in adipose tissues [6]. In the liver, the physiological function of PPARγ is less clear, although its expression is associated with injury-induced activation of hepatostellate cells and provides an anti-fibrogenic protection [3, 5]. PPARγ up-regulation is also a known property of steatotic livers, and liver-specific disruption of PPARγ was reported to protect leptin-deficient mice or HFD-fed mice from developing fatty liver [7–9]. Here, we show that mice deficient for HECT-domain E3 ligase Smurf1 in the mixed BL genetic background develop hepatosteatosis spontaneously as they age or are more susceptible to HFD-induced hepatosteatosis. These mutant mice are overweight and obese, as well as glucose intolerant and insulin resistant. These NAFLD phenotypes can be attributed to the heightened transcriptional activity of PPARγ, which in turn increases the expression of itself and genes involved in lipogenesis and fatty acid transport via a positive feedback loop. We further demonstrate that Smurf1 catalyzes the K63-linked non-proteolytic ubiquitination that normally attenuates PPARγ transcriptional activity, and show an inverse correlation between low SMURF1 expression and high BMI values in human patients. This investigation thus reveals a previously unknown mechanism that regulates the lipogenic activity of PPARγ and sheds light on a new role of Smurf1 in NAFLD pathogenesis.

Different HECT E3 ligases are known to catalyze ubiquitination with different ubiquitin chains that mark modified protein substrates for different fates [38]. Members of the neural precursor cell expressed developmentally down-regulated protein 4 (NEDD4) family E3 ligases preferentially support monoubiquitin modification or K63-linked chains associated with non-proteolytic functions, but can also assemble lysine 48 (K48)-linked chains that target proteins for proteasome-mediated degradation [39]. As members of this E3 ligase family, Smurf1 and Smurf2 have been shown to target many proteins for K48-linked ubiquitination and degradation [16]. Smurf2 was also shown to induce multi-monoubiquitin modification of Smad3, thereby inhibiting Smad3 activity [40], but the K63-linked ubiquitination by Smurfs has not been reported in mammalian species. Recently, NEDD4 itself was shown to induce both K48- and K63-linked ubiquitination of PPARγ in adipocytes, with different functional outcomes [41]. In our study, Smurf1 inhibits PPARγ activity, and deletion of Smurf1 enhances PPARγ activity and up-regulates PPARγ levels through a positive feedback mechanism. In contrast, NEDD4 was shown to stabilize PPARγ, and knockdown of NEDD4 reduced PPARγ expression [41]. Moreover, the PY motif in PPARγ played a role in mediating interaction with Smurf1, but it was not demonstrated for NEDD4 as such. Perhaps these apparent discrepancies reflect the differences in experimental conditions conducted in different cell types, or the mixed linkages in ubiquitin chains formed by NEDD4 could have compounded the functions of modified PPARγ. In any event, the steatosis observed in Smurf1KO mice is consistent with the heightened PPARγ activity in the liver. Despite the conspicuous steatosis, overweight, and obesity that were present in 75% aged BL-Smurf1KO mice, their liver functions were nevertheless normal. Because these animals were well shielded from inflammatory insults by their accommodative housing facility, it is likely that the elevated PPARγ activity unleashed by the loss of Smurf1 was only sufficient to manifest a restricted impact in bringing about the early-stage NAFLD phenotypes. Future studies are necessary to ascertain the tissue origin of the steatogenic effect of Smurf1 ablation using conditional knockout approaches and to determine if and how BL-Smurf1 mice could be enticed to progress through NASH or even liver cancer to model the entire NAFLD disease spectrum. Regulation of PPARγ by the Smurf1-mediated K63-linked ubiquitin modification centers on its transcriptional activity. Because PPARγ is also a transcriptional target of its own, a disturbance of Smurf1 would create an “all or none” effect: a rise or fall of Smurf1 across a threshold level would either maximize or minimize PPARγ activity. This scenario may normally operate to keep the lipogenic activity of PPARγ to a minimum in the liver but maximized in the adipose tissues.

Epidemiology studies indicate that an estimated 27%–34% of the general population within North America have NAFLD [42], for which there is no approved treatment available at present. Current NAFLD drug developmental effort centers on repurposing fibric acid derivatives, which are lipid-lowering PPARα agonists and insulin sensitivity–improving PPARγ agonists, thiazolidinediones, but the clinical trials yielded mixed results [3, 4]. Because of the opposite actions of PPARα and PPARγ on hepatic steatosis, the “spillover” effects of these PPAR agonists might prevent a net gain in their ability to reduce TG accumulation in the liver. As to PPARγ agonists, although clinical trials for rosiglitazone in patients with type 2 diabetes reported improvement of steatosis by a median of 20% during the first year, no further improvement was found after 2 additional years of treatment, and the trials exposed severe cardiovascular risks and weight gain [43]. Intuitively, it is possible that the benefit is derived from the systemic lipid clearance by increased fat storage in adipose tissue, because PPARγ is normally expressed in adipose tissues, and its activation in the liver was clearly linked to fatty liver formation. Given our current finding of Smurf1 in protecting the liver from steatosis, a viable strategy to treat NAFLD may be to curtail the transcriptional activity of PPARγ by turning on Smurf1-mediated non-proteolytic ubiquitin modification.

## Materials and methods

### Ethics statement

All mice were maintained and handled under protocols (LCMB-014, ASP 10–214, 13–214, 16–214) approved by the Animal Care and Use Committee of the National Cancer Institute, National Institutes of Health (NIH), according to NIH guidelines.

### Animals and treatment

Generation of Smurf1KO and Smurf2KO mice in the mixed BL and pure C57BL/6N (B6) background was described previously [18, 40]. For spontaneous hepatosteosis development, animals were maintained on a ND, monitored weekly, and euthanized and necropsied at 9–12 months of age. For the HFD treatment, male mice were maintained on a ND until 10–12 weeks of age before they were given HFD (Research Diets, Cat# D12266B) containing 16.8% kcal protein, 31.8% kcal fat, and 51.4% kcal carbohydrate for 8 weeks. For the GW9662 treatment, a dose of 1 mg/kg of GW9662 dissolved in DMSO was injected intraperitoneally (i.p.) into 7–9-month-old BL-WT and BL-Smurf1KO mice for 5 days per week for 2 months. Age and sex of mice used in these studies are listed in S1 Table.

### Measurement of body composition and histology

Body composition was determined using an EchoMRI mouse scanner (EchoMRI, Houston, TX). Mouse liver and epididymal fat pad were dissected, weighed, then either snap-frozen in liquid N_2_ or fixed in 10% neutral buffered formalin prior to paraffin embedding. Frozen liver tissues were used for Oil Red-O staining. Liver and fat tissue histology were read by board-certified veterinary pathologists in the Pathology and Histotechnology Laboratory of the Frederick National Laboratory for Cancer Research.

### Blood and liver biochemical analysis

Serum TG, CHO, and albumin concentrations, as well as ALT and AST activities were measured by standard methods with a Vitro 250 dry slide analyzer (Ortho Clinical Diagnostics) in the Pathology and Histotechnology Laboratory of the Frederick National Laboratory for Cancer Research. Liver TG, CHO, and FFA concentrations were determined using the EnzyChrom TG, CHO, and FFA assay kits (Bioassay Systems) after extracting total lipids from 50-mg liver tissues as described [44].

### Glucose tolerance test and insulin tolerance test

To perform the gluclose tolerance test (GTT) or insulin tolerance test (ITT), mice were fasted overnight before receiving an i.p. injection of 20% glucose (2 g/kg body weight) or recombinant insulin (Humulin R, 0.75 U/kg; Lily), respectively. Blood samples were collected from the tail 0, 0.5, 1, 2, and 4 hours later, after injection for analysis using the Accu-Chek Compact Plus blood glucose meter (Roche Diagnostics).

### Cell culture, plasmids, antibodies, and reagents

AML12 cells (ATCC CRL-2254) were cultured in DMEM/F12 supplemented with 10% fetal bovine serum (FBS), 0.005 mg/mL insulin, 0.005 mg/mL transferrin, 5 ng/mL selenium, and 40 ng/mL dexamethasone. Hep3B cells were cultured in MEM supplemented with 1% Non-Essential Amino Acids (NEAA) and 10% FBS. Smurf1KO MEFs were cultured in DMEM supplemented with 10% FBS. Primary hepatocytes were isolated by a two-step collagenase perfusion of the liver and cultured as described [45]. Flag-tagged PPARγ1, PPARγ2 plasmids, and PPRE-Luc reporter plasmids were obtained from Addgene. Flag-tagged PPARγ2ΔPY plasmid was generated using Site Directed Mutagenesis Kit (Agilent Technologies). Myc-tagged Smurf1, Smurf2, and Smurf1CA mutant, HA-tagged Ubiquitin plasmids were described before [13, 18, 46]. Anti-Smurf1 (Novus, 1D7); anti-Smurf2 (Abcam, EP629Y3); anti-PPARγ (Santa Cruz, sc-7273); anti-PPARα (Rockland, 600-401-4215); anti-PPARδ (ThermoFisher, PA1-823A); anti-HSC70 (Santa Cruz, B-6); Anti-Flag-Peroxidase (A8592, Sigma); anti-HA (Covance, HA11); and anti-Myc (Santa Cruz, 9E10) were used for western blotting and immunoprecipitation. Knockdown experiments were performed using the following siRNAs: siPPARγ (J-040712-05 and J-040712-07, Dharmacon). Validated siSmurf1, siSmurf2 and siNS were previously described [47, 48].

### Lipid synthesis and fatty acid uptake

Lipogenesis assay in AML12 cells and primary hepatocytes were performed using ^3^H-acetate as described [45]. Fatty acid uptake assay in AML12 cells was performed in 12-well plates. Briefly, AML12 cells were incubated with assay buffer (Hanks’ balanced buffer containing 1% BSA and 5 μCi/mL ^3^H-palmitic acid) for 60 minutes at 37°C. The cells were then washed twice with ice-cold PBS and lysed with 0.3 M NaOH. The radioactivity of the cell lysates was measured by liquid scintillation counting. In vivo fatty acid uptake assays were performed as described [49]. Briefly, mice were i.p. injected with BODIPY-FL-C16 (Life Technologies) after being fasted for 4 hours, then were euthanized at 5 hours after injection; liver, epididymal fat pad, and skeletal muscle were collected. Fluorescence was analyzed from cleared tissue homogenate using a plate reader and normalized to tissue weight.

### Differention of 3T3-L1 cells into adipocytes

Preadipocytes 3T3-L1 (ATCC, CL-173) were cultured in basal medium (DMEM supplemented with 10% FBS). Two days after transfection with siRNA, basal media were changed to differentiation media (day 0), which is DMEM supplemented with 10% FBS, 0.5 mM IBMX, 1μM dexamethasone, and 4 μg/mL insulin, for 2 days, then replaced with basal media with 2 μg/mL insulin for another 4 days. After 6 days of differentiation, cells were harvested for protein and mRNA analysis or subjected to Oil Red staining.

### In vitro ubiquitination assays

The purified recombinant PPARγ (0.25 μg) (Abcam, ab81807) and His6-Smurf1 (1.5 μg) were used in in vitro ubiquitination assay, which was carried out for 1 hour at 37°C in 30 μl reaction buffer supplemented with 2 mM Mg-ATP, 1 μg E1, 1 μg of recombinant UbcH5c, and 20 μg HA-ubiquitin or HA-ubiquitin variants (all from Boston Biochem).

### RNA extraction and qRT-PCR

Total RNA from AML12 cells or liver tissues was extracted by RNeasy Mini Kit (Qiagen) according to the manufacturer’s instructions. High Capacity Reverse Transcription Kit (ABI, Life Tech) was used to generate cDNA from RNA (500–2,000 ng). qRT-PCR was performed with Power SYBR Green PCR Master Mix (Life Technologies) using specific oligonucleotide primers as specified (S4 Table).

### ChIP and luciferase reporter assays

ChIP assays were carried out with an EZ-ChIP Chromatin Immunoprecipitation Kit (Millipore) according to the manufacturer’s instructions. Immunoprecipitations were carried out using anti-PPARγ antibody (Abcam, A3409A) and an isotype-matched IgG as the control. Reporter assays were performed in 12-well plates using PPRE-Luc (0.5 μg) and pRL-TK (0.2 μg) reporter plasmids, and the luciferase activities were determined using Dual Luciferase Reporter Assay System (Promega).

### Microarray, KEGG pathway, and metabolomic profile analysis

Microarray experiments for mouse liver tissues were performed on Affymetrix GeneChip Mouse Gene 1.0 ST arrays according to the standard Affymetrix GeneChip protocol at the Affymetrix service core in the Frederick National Laboratory for Cancer Research. The raw array data were then analyzed with packages oligo and lima under R platform, as described before [50, 51], to identify differentially expressed genes among groups (fold > 1.5, FDR *cutoff = 0*.*1*), and results were visualized using VennDiagram (https://cran.r-project.org/web/packages/VennDiagram) and gplots (https://cran.r-project.org/web/packages/gplots) under R platform. Data were submitted to GEO (accession number GSE113995). KEGG pathway analysis was performed by gage package, as described [52], to identify significantly enriched pathway (FDR *q-value cutoff = 0*.*1*) between Smurf1KO and WT liver samples.

The microarray analysis for human liver tissues from the LCI cohort of 247 Chinese patients was previously published [22] and data are accessible through GEO (accession number GSE14520). TCGA non-tumor liver tissue gene expression data were downloaded from TCGA-LIHC (https://portal.gdc.cancer.gov).

### Statistical analysis

Unless indicated in the figure legends, two-tailed Student *t* test was used for statistical analysis.

## Supporting information

S1 Fig Glucose and insulin tolerance tests in BL-WT and BL-Smurf1KO mice at age 4–5 months. **(A)** Glucose and **(B)** insulin tolerance tests in male WT and Smurf1KO (SF1KO) mice at age 4–5 months (*n* = 8 per group). All data are presented as mean ± SD; statistical significance of differences is indicated as *p < 0.05, **p < 0.01, ***p < 0.001. Original raw data can be found in S1 Data. BL, mixed black Swiss × 129/SvEv background; KO, knockout; SF1KO, Smurf1KO; Smurf, Smad ubiquitin regulatory factor; WT, wild-type.
(TIF)

S2 Fig Ablation of Smurf1 exacerbates HFD-induced steatosis in mice of the C57BL/6 (B6) background. **(A)** BW, Fat/Lean and Liver/BW ratios, and histological scores of steatosis in male mice from B6 background reared on either a ND or HFD beginning at 10–12 weeks of age for 8 weeks. WT, *n* = 8 per group; Smurf1KO (SF1KO), *n* = 7 per group. **(B)** HE staining of representative liver sections of the above B6 mice at the end of diet treatment. Bar = 100 μm. **(C)** Liver TG levels of the above B6 mice. Data are presented as mean ± SD; statistical significance of differences is indicated as *p < 0.05, **p < 0.01, ***p < 0.001. Original raw data can be found in S1 Data. BW, body weight; Fat/Lean, fat mass to lean mass; HE, hematoxylin–eosin; HFD, high-fat diet; Liver/BW, liver weight to body weight; ND, normal diet; ns, not significant; SF1KO, Smurf1KO; Smurf, Smad ubiquitin regulatory factor; TG, triglyceride.
(TIF)

S3 Fig Up-regulation of PPARγ associated with Smurf1 loss. **(A)** qRT-PCR analyses of total *Ppar*γ in livers from BL-WT and Smurf1KO male mice from BL background reared on either a ND (*n* = 7 per group) or HFD (*n* = 6 per group) beginning at 10–12 weeks of age for 8 weeks. **(B)** qRT-PCR analyses of *Tnf*α and F4/80 in livers of the above mice. Data are presented using box and whisker plot showing all points; centerline represents the median, and statistical significance of differences between WT and Smurf1KO is indicated as *p < 0.05, **p < 0.01, and ***p < 0.001. Original raw data can be found in S1 Data. BL, mixed black Swiss × 129/SvEv background; HFD, high-fat diet; KO, knockout; ND, normal diet; PPAR, peroxisome proliferator-activated receptor; qRT-PCR, quantitative real-time PCR; Smurf, Smad ubiquitin regulatory factor; WT, wild-type.
(TIF)

S4 Fig Smurf1 had no effect on *Cebpα/β* mRNA expression and siRNA knockdown efficiency in AML12 cells. **(A)** qRT-PCR analyses showing that knockdown of Smurf1 had no effect on *Cebpα/β* mRNA in AML12 cells. **(B)** qRT-PCR analyses showing knockdown efficiency of siSmurf1 and siPparγ in AML12 cells. Data are presented as mean ± SD; statistical significance of differences is indicated as **p < 0.01, ***p < 0.001. Original raw data can be found in S1 Data. *Cebpα/β*, CCAAT enhancer binding protein *α* or *β*; qRT-PCR, quantitative real-time PCR; siRNA, short interfering RNA; Smurf, Smad ubiquitin regulatory factor.
(TIF)

S5 Fig Smurf1 regulates adipogenesis through PPARγ. **(A)** Western blot analyses of siRNA transfected 3T3-L1 cells at the beginning or after 6 days of differentiation. Double: cells were transfected with both siSmurf1 and siPparγ. **(F)** Oil-Red staining of the above siRNA-transfected 3T3-L1 cells after differentiation for 6 days. **(G)** qRT-PCR analyses showing the up-regulation of a group of lipogenic and PPARγ target genes in siRNA-transfected 3T3-L1 cells after 6 days of differentiation, *n* = 3. Data are presented as mean ± SD; statistical significance of differences is indicated as *p < 0.05, **p < 0.01, ***p < 0.001. Original raw data can be found in S1 Data. PPAR, peroxisome proliferator-activated receptor; qRT-PCR, quantitative real-time PCR; siRNA, short interfering RNA; Smurf, Smad ubiquitin regulatory factor.
(TIF)

S1 Table Age and sex of mice used in different experiments. (PDF)

S2 Table Tissue weights and blood parameters in mice fed with HFD. HFD, high-fat diet.
(PDF)

S3 Table Differentially expressed gene lists. (1) Differentially expressed genes in Smurf1KO livers compared with that of WT livers from BL-background mice at age 11 months. (2) Differentially expressed genes in Smurf2KO livers compared with that of WT livers from BL-background mice at age 11 months. BL, xxx; KO, knockout; Smurf, Smad ubiquitin regulatory factor; WT, wild-type.
(XLSX)

S4 Table Primer sequences. (PDF)

S1 Data Underlying numeric data used in this work. (XLSX)

## Abbreviations

ALT: alanine transaminase
AST: aspartate transaminase
AUC: area under the curve
BL: mixed black Swiss × 129/SvEv background
BMI: body mass index
BMP: bone morphogenetic protein
BODIPY-FL-C16: 4,4-Difluoro-5,7-Dimethyl-4-Bora-3a,4a-Diaza-s-Indacene-3-Hexadecanoic Acid
B6: C57BL/6N
CEBP: CCAAT enhancer binding protein
ChIP: chromatin immunoprecipitation
CHO: total cholesterol
E3: ubiquitin ligase
FDR: false discover rate
FFA: free fatty acid
HE: hematoxylin–eosin
HECT: homologous to E6-AP carboxyl terminus
HFD: high-fat diet
Hsc70: heat shock cognate 71 kDa protein
KEGG: Kyoto Encyclopedia of Genes and Genomes
KO: knockout
K48: lysine 48
K63: lysine 63
IB: immunoblot
LCI: Liver Cancer Institute
MEF: mouse embryonic fibroblast
NAFLD: nonalcoholic fatty liver disease
NASH: nonalcoholic steatohepatitis
ND: normal diet
NEDD4: neural precursor cell expressed developmentally down-regulated protein 4
PPAR: peroxisome proliferator-activated receptor
PPARγ: peroxisome proliferator-activated receptor γ
PPRE: PPAR response element
PY: PPxY
qRT-PCR: quantitative real-time PCR
Rxr: retinoid x receptor
siRNA: short interfering RNA
siNS: non-silencing control siRNA
Smurf: Smad ubiquitin regulatory factor
SUMO: small ubiquitin-like modifier
TCGA-LIHC: the cancer genome atlas-liver hepatocellular carcinoma
TG: triglyceride
TGF-β: transforming growth factor-β
WAT: white adipose tissue
WT: wild-type
