# Supplementary figures and images for: Non-proteolytic ubiquitin modification of PPARγ by Smurf1 protects the liver from steatosis
Source: PLoS Biol. 2018 Dec 19;16(12):e3000091. doi: 10.1371/journal.pbio.3000091 (PMC6317813; doi:10.1371/journal.pbio.3000091)

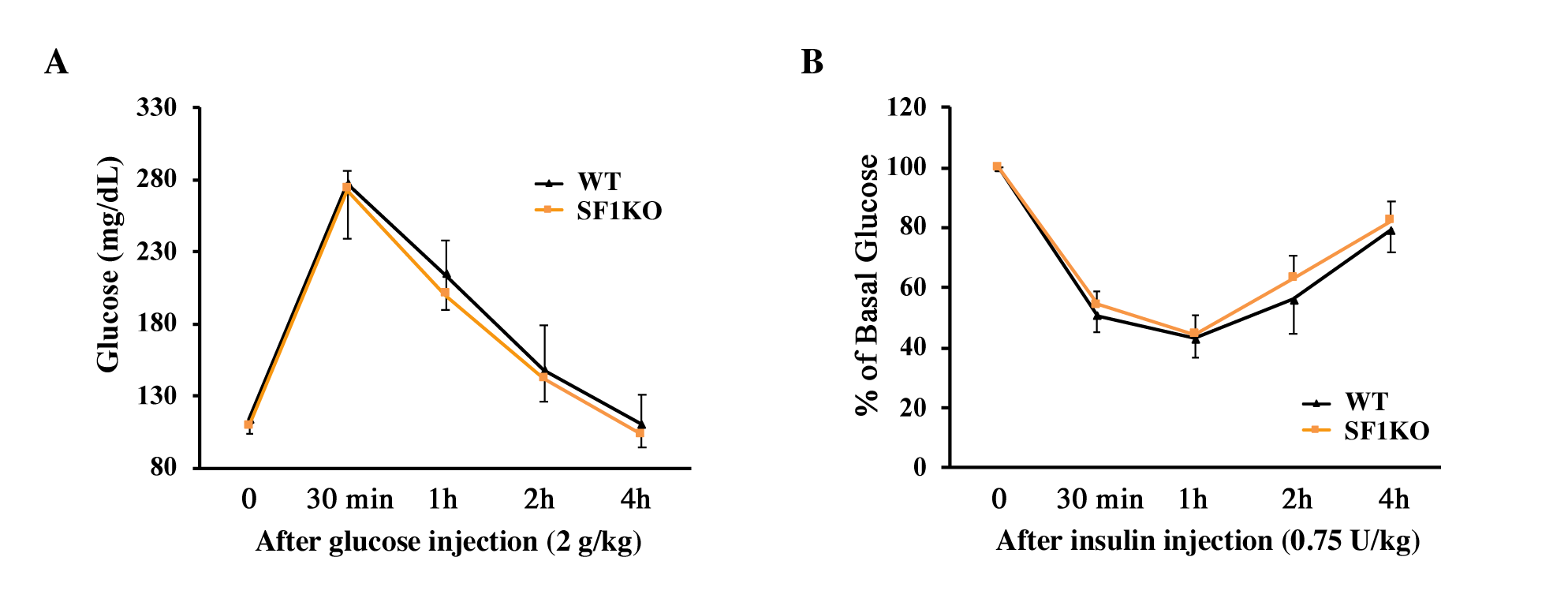

Supplement: S1 Fig — (A) Glucose and (B) insulin tolerance tests in male WT and Smurf1KO (SF1KO) mice at age 4–5 months (n = 8 per group). All data are presented as mean ± SD; statistical significance of differences is indicated as *p < 0.05, **p < 0.01, ***p < 0.001. Original raw data can be found in S1 Data. BL, mixed black Swiss × 129/SvEv background; KO, knockout; SF1KO, Smurf1KO; Smurf, Smad ubiquitin regulatory factor; WT, wild-type. (TIF) [file pbio.3000091.s001.tif]

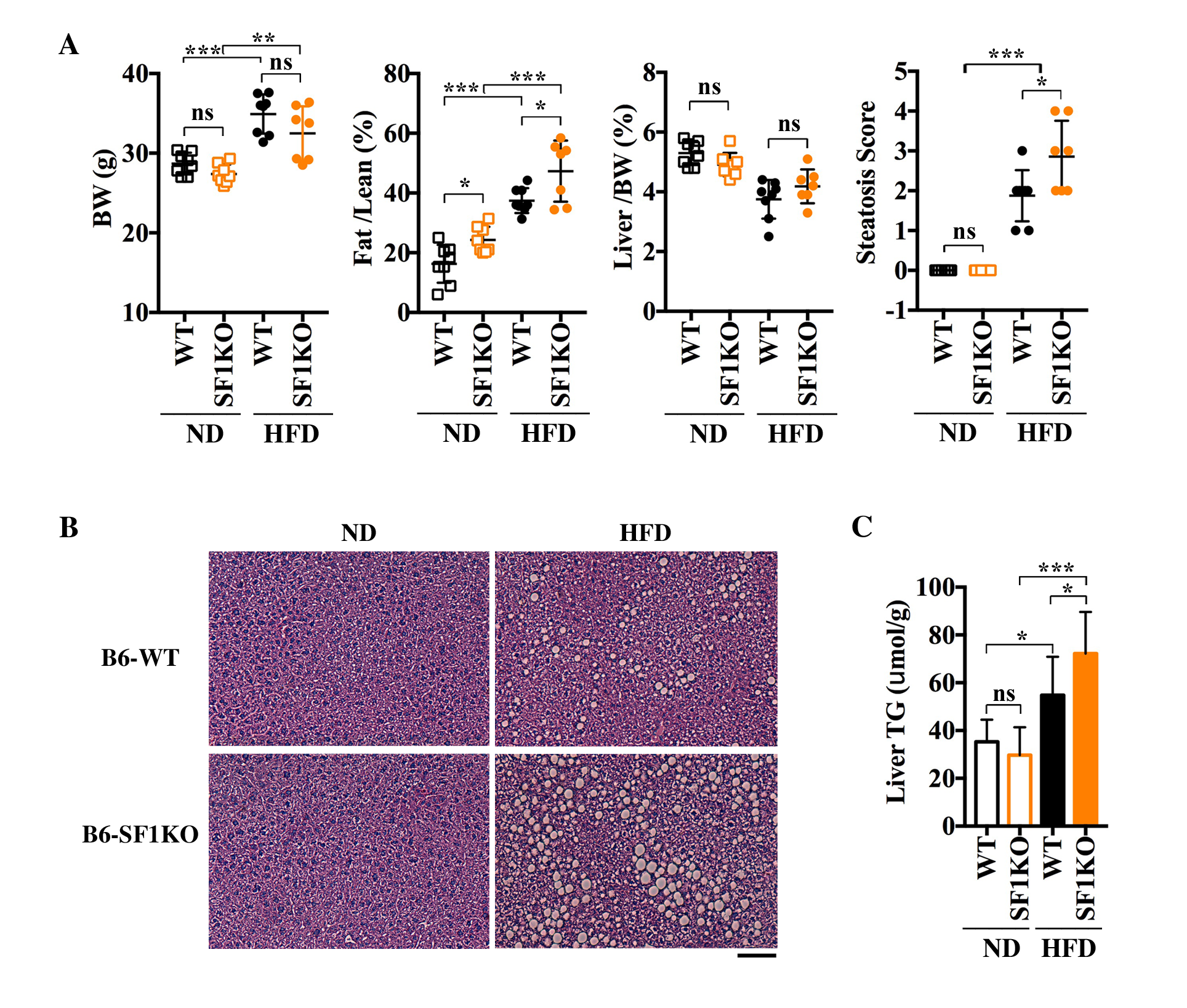

Supplement: S2 Fig — (A) BW, Fat/Lean and Liver/BW ratios, and histological scores of steatosis in male mice from B6 background reared on either a ND or HFD beginning at 10–12 weeks of age for 8 weeks. WT, n = 8 per group; Smurf1KO (SF1KO), n = 7 per group. (B) HE staining of representative liver sections of the above B6 mice at the end of diet treatment. Bar = 100 μm. (C) Liver TG levels of the above B6 mice. Data are presented as mean ± SD; statistical significance of differences is indicated as *p < 0.05, **p < 0.01, ***p < 0.001. Original raw data can be found in S1 Data. BW, body weight; Fat/Lean, fat mass to lean mass; HE, hematoxylin–eosin; HFD, high-fat diet; Liver/BW, liver weight to body weight; ND, normal diet; ns, not significant; SF1KO, Smurf1KO; Smurf, Smad ubiquitin regulatory factor; TG, triglyceride. (TIF) [file pbio.3000091.s002.tif]

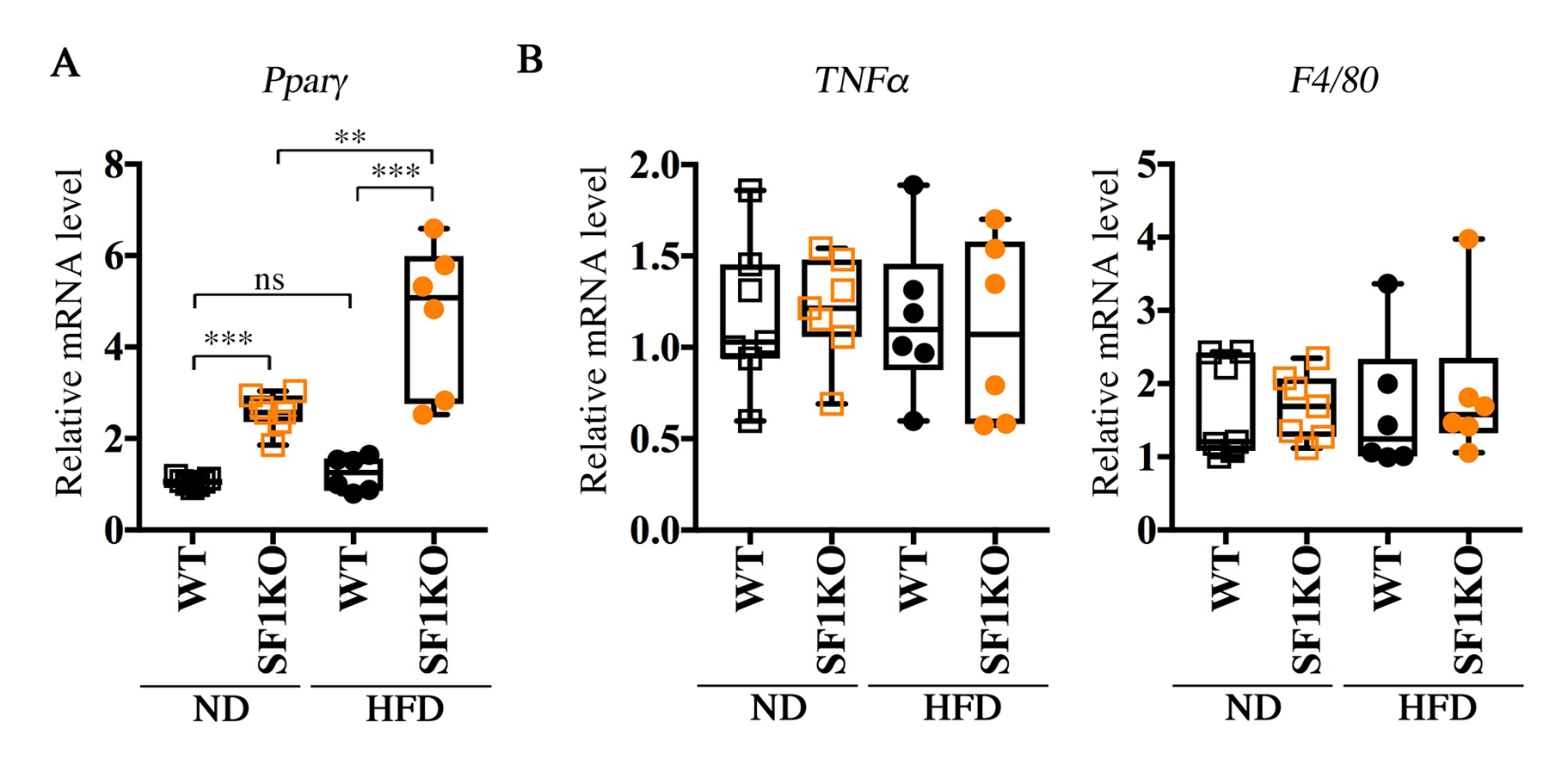

Supplement: S3 Fig — (A) qRT-PCR analyses of total Pparγ in livers from BL-WT and Smurf1KO male mice from BL background reared on either a ND (n = 7 per group) or HFD (n = 6 per group) beginning at 10–12 weeks of age for 8 weeks. (B) qRT-PCR analyses of Tnfα and F4/80 in livers of the above mice. Data are presented using box and whisker plot showing all points; centerline represents the median, and statistical significance of differences between WT and Smurf1KO is indicated as *p < 0.05, **p < 0.01, and ***p < 0.001. Original raw data can be found in S1 Data. BL, mixed black Swiss × 129/SvEv background; HFD, high-fat diet; KO, knockout; ND, normal diet; PPAR, peroxisome proliferator-activated receptor; qRT-PCR, quantitative real-time PCR; Smurf, Smad ubiquitin regulatory factor; WT, wild-type. (TIF) [file pbio.3000091.s003.tif]

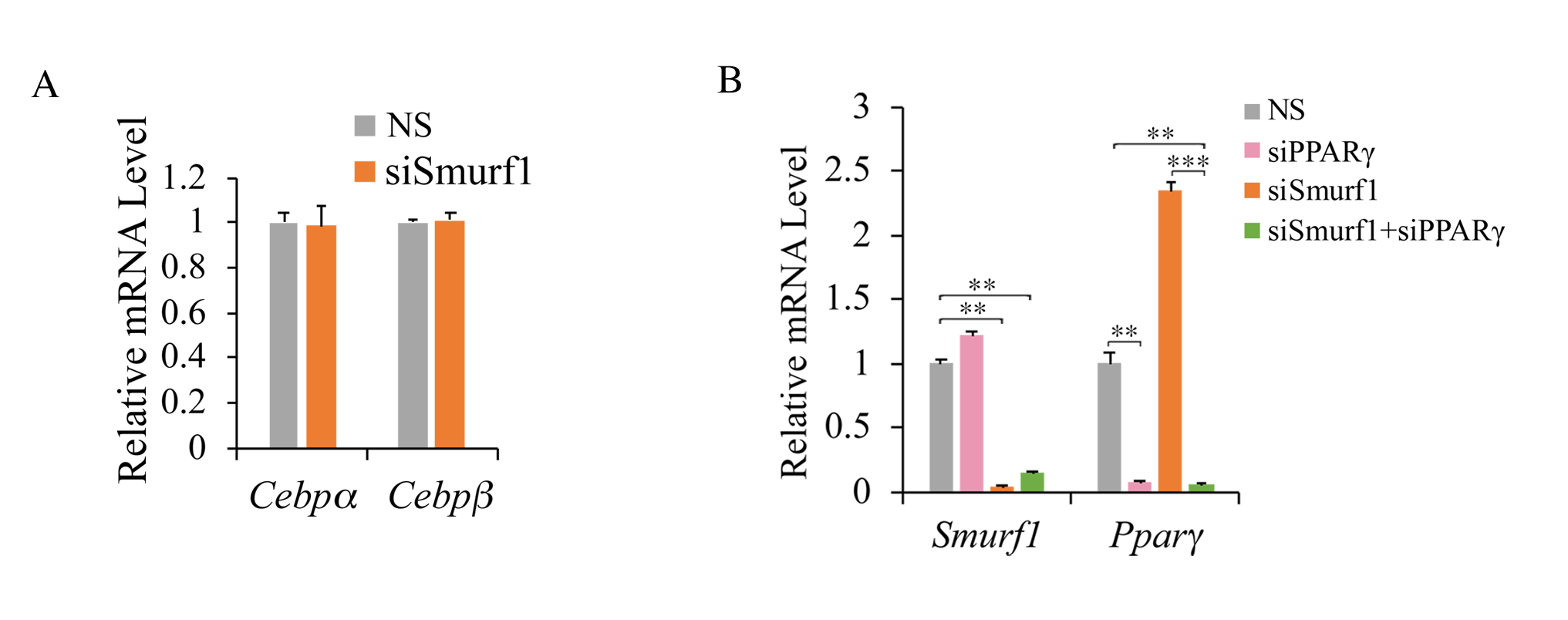

Supplement: S4 Fig — (A) qRT-PCR analyses showing that knockdown of Smurf1 had no effect on Cebpα/β mRNA in AML12 cells. (B) qRT-PCR analyses showing knockdown efficiency of siSmurf1 and siPparγ in AML12 cells. Data are presented as mean ± SD; statistical significance of differences is indicated as **p < 0.01, ***p < 0.001. Original raw data can be found in S1 Data. Cebpα/β, CCAAT enhancer binding protein α or β; qRT-PCR, quantitative real-time PCR; siRNA, short interfering RNA; Smurf, Smad ubiquitin regulatory factor. (TIF) [file pbio.3000091.s004.tif]

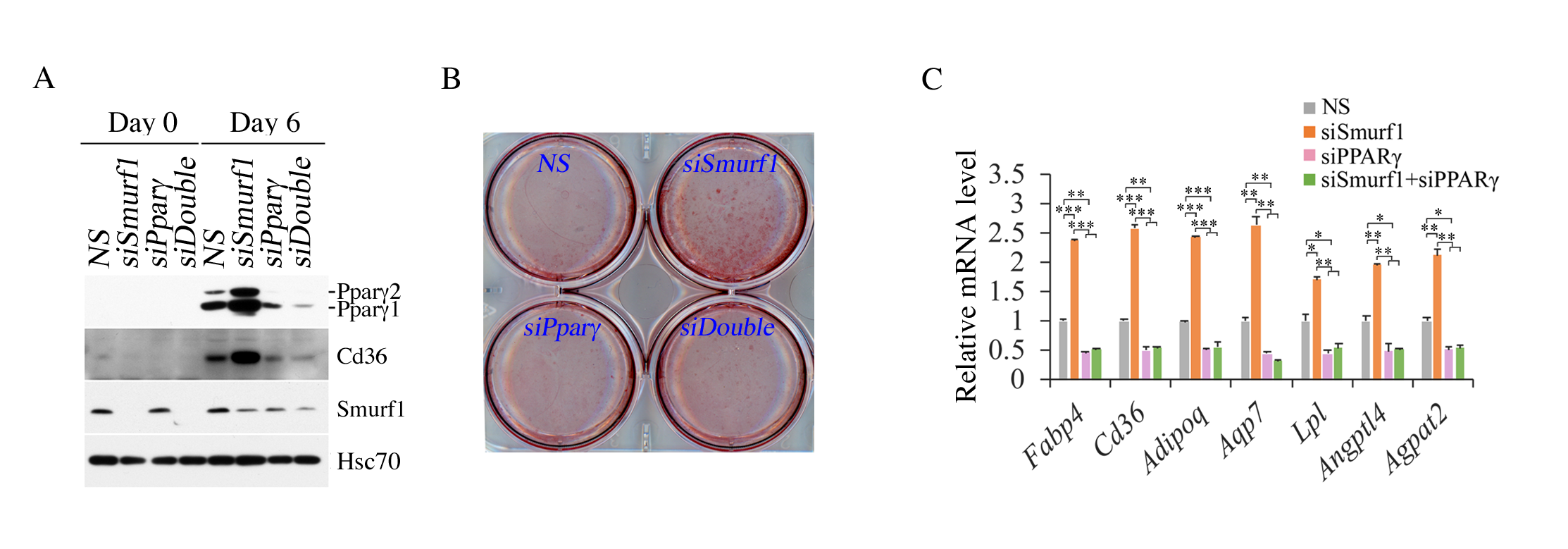

Supplement: S5 Fig — (A) Western blot analyses of siRNA transfected 3T3-L1 cells at the beginning or after 6 days of differentiation. Double: cells were transfected with both siSmurf1 and siPparγ. (F) Oil-Red staining of the above siRNA-transfected 3T3-L1 cells after differentiation for 6 days. (G) qRT-PCR analyses showing the up-regulation of a group of lipogenic and PPARγ target genes in siRNA-transfected 3T3-L1 cells after 6 days of differentiation, n = 3. Data are presented as mean ± SD; statistical significance of differences is indicated as *p < 0.05, **p < 0.01, ***p < 0.001. Original raw data can be found in S1 Data. PPAR, peroxisome proliferator-activated receptor; qRT-PCR, quantitative real-time PCR; siRNA, short interfering RNA; Smurf, Smad ubiquitin regulatory factor. (TIF) [file pbio.3000091.s005.tif]
