# Supplementary material for: Non-proteolytic ubiquitin modification of PPARγ by Smurf1 protects the liver from steatosis
Source: PLoS Biol. 2018 Dec 19;16(12):e3000091. doi: 10.1371/journal.pbio.3000091 (PMC6317813; doi:10.1371/journal.pbio.3000091)
Supplement: S1 Table — (PDF) [file pbio.3000091.s006.pdf]

S1 Table: Age, sex and steatosis of mice used in different experiments

	Spontaneous hepatosteatosis				
	BL-WT	BL-SF1KO	BL-SF2KO	B6-WT	B6-SF1KO
Number of mice					
Male; female	7; 8	7; 8	7; 8	5; 5	5; 5
Age (month)					
Range	9-12	9-12	9-12	11-12	11-12
Liver Steatosis*	2/15	12/15	3/15	0/10	0/10
Liver Inflammation**	0/15	0/15	0/15	0/10	0/10
	GTT and ITT				
	BL-WT	BL-SF1KO			
Number of mice					
Male	8 + 8	8 + 8			
Age (month)					
Range	9-12 (n=8)	9-12 (n=8)			
Range	4.5-5 (n=8)	4.5-5 (n=8)			
	HFD treatment (BL-mice)				
	WT(ND)	SF1KO(ND)	WT(HFD)	SF1KO(HFD)	
Number of mice					
Male	7	7	8	8	
Age (month)					
Range (starting)	2.5-3	2.5-3	2.5-3	2.5-3	
Range (end)	4.5-5	4.5-5	4.5-5	4.5-5	
Liver Steatosis*	0/7	0/7	5/7	6/7	
Inflammation**	0/7	0/7	0/8	0/8	
	HFD treatment (B6-mice)				
	WT(ND)	SF1KO(ND)	WT(HFD)	SF1KO(HFD)	
Number of mice					
Male	8	7	8	7	
Age (month)					
Range (starting)	2.5-3	2.5-3	2.5-3	2.5-3	
Range (end)	4.5-5	4.5-5	4.5-5	4.5-5	
Liver Steatosis*	0/8	0/7	5/7	7/7	
	GW9662 treatment (BL-mice)				
	WT(GW)	SF1KO (GW)	WT (Ctrl)	SF1KO(Ctrl)	
Number of mice					
Male	8	8	8	8	
Age (month)					
Range (starting)	7-9	7-9	7-9	7-9	
Range (end)	9-12	9-12	9-12	9-12	
Liver Steatosis*	0/8	0/8	0/8	7/8	

Steatosis: NO: < 10% steatosis, score 0 or 1; Yes: >10% steatosis, score 2, 3, 4.

Inflammation: NO: score 0 or 1; Yes, score 2, 3, 4.
