# Supplementary material for: Non-proteolytic ubiquitin modification of PPARγ by Smurf1 protects the liver from steatosis
Source: PLoS Biol. 2018 Dec 19;16(12):e3000091. doi: 10.1371/journal.pbio.3000091 (PMC6317813; doi:10.1371/journal.pbio.3000091)
Supplement: S2 Table — HFD, high-fat diet. (PDF) [file pbio.3000091.s007.pdf]

S2 Table, related to Figure 2:

Tissue weights, food consumption and blood parameters in mice fed with HFD

	BL		B6	
	WT (n=8)	SF1KO (n=8)	WT (n=8)	SF1KO (n=7)
Body weight, g	26.73±2.43	31.76±2.80**	34.93±2.47	32.48±3.40
Food consumption, g/d	2.40±0.17	2.51±0.11	2.91±0.2	2.79±0.15
Fat mass, g	3.90±1.03	7.13±2.02**	9.28±1.11	10.29±2.57
Lean mass, g	21.57±1.91	23.03±1.45	24.77±1.03	21.60±1.16**
Fat mass/Lean mass, %	18.15±4.75	30.92±8.33**	37.44±4.18	47.32±10.21*
Liver weight, g	1.06±0.15	1.40±0.39*	1.30±0.18	1.36±0.20
Liver weight/Body weight, %	3.96±0.44	4.37±0.97	3.75±0.63	4.19±0.55
Serum FFA, µmol/dL	28.42±15.65	44.41±10.62*	28.71±6.36	23.59±1.74
Serum TG, mg/dL	146.2±30.9	161.9±34.1	106.4±22.0	136.5±31.1*
Serum Chloesterol, mg/dL	192.7±17.2	193.9±26.1	142.1±42.8	175.1±17.2
AST, U/L	155.1±102.4	158.9±94.6	211.8±113.4	237.4±114.7
ALT, U/L	43.5±13.9	65.9±18.3**	96.1±66.2	95.1±37.0
Albumin, g/dL	2.9±0.2	2.7±0.2*	3.0±0.4	3.5±0.4*

Data represents mean ± SD, * $p < 0.05$, **$p<0.02$ compared with WT.
