# Supplementary material for: Non-proteolytic ubiquitin modification of PPARγ by Smurf1 protects the liver from steatosis
Source: PLoS Biol. 2018 Dec 19;16(12):e3000091. doi: 10.1371/journal.pbio.3000091 (PMC6317813; doi:10.1371/journal.pbio.3000091)
Supplement: S4 Table — (PDF) [file pbio.3000091.s009.pdf]

S4 Table: Primer sequences.

Name	Seqence	Application
PpargΔPY-F	AAAGTAGAACCTGCATCTTCTGAAAAGACCCAGCTC	Mutagenesis
PpargΔPY-R	GAGCTGGGTCTTTTCAGAAGATGCAGGTTCTACTTT	
Pparg1-F	AAGAAGCGGTGAACCACTGA	RT-qPCR
Pparg1-R	GAATGCGAGTGGTCTTCCAT	
Pparg2-F	TCGCTGATGCACTGCCTATG	
Pparg2-R	GAGAGGTCCACAGAGCTGATT	
Pparg-F	CTCCAAGAATACCAAAGTGCGA	
Pparg-R	GCCTGATGCTTTATCCCCACA	
Ppara-F	TACTGCCGTTTTCACAAGTGC	
Ppara-R	AGGTCGTGTTCACAGGTAAGA	
Ppard-F	TCCATCGTCAACAAAGACGGG	
Ppard-R	ACTTGGGCTCAATGATGTCAC	
Rxra-F	ATGGACACCAAACATTTCCTGC	
Rxra-R	CCAGTGGAGAGCCGATTCC	
Rxrb-F	CCACCTCTTACCCCTTCAGC	
Rxrb-R	TGGAAGAACTGATGACTGGGA	
Cebpd-F	CGACTTCAGCGCCTACATTGA	
Cebpd-R	CTAGCGACAGACCCCACAC	
Cebpa-F	GCGGGAACGCAACAACATC	
Cebpa-R	GTCACTGGTCAACTCCAGCAC	
Cebpb-F	CAAGCTGAGCGACGAGTACA	
Cebpb-R	AGCTGCTCCACCTTCTTCTG	
Acacb-F	CGCTCACCAACAGTAAGGTGG	
Acacb-R	GCTTGGCAGGGAGTTCCTC	
Cpt1a-F	CTCCGCCTGAGCCATGAAG	
Cpt1a-R	CACCAGTGATGATGCCATTCT	
Cyp4a12b-F	GGGGAGATCAGACCCAAAAGC	
Cyp4a12b-R	ATTCGTCGGTGCTGAAACCAT	
Cyp8b1-F	CTAGGGCCTAAAGGTTCGAGT	
Cyp8b1-R	GTAGCCGAATAAGCTCAGGAAG	
Elovl6-F	GAAAAGCAGTTCAACGAGAACG	
Elovl6-R	AGATGCCGACCACCAAAGATA	
Fads2-F	GATGGCTGCAACATGACTATGG	
Fads2-R	GCTGAGGCACCCTTTAAGTGG	
Scd1-F	TTCTTGCGATACACTCTGGTGC	
Scd1-R	CGGGATTGAATGTTCTTGTCGT	
Apoa2-F	TGGTCGCACTGCTGGTAAC	
Apoa2-R	TTTGCCATATTCAGTCATGCTCT	
Apoc3-F	TACAGGGCTACATGGAACAAGC	
Apoc3-R	CAGGGATCTGAAGTGATTGTCC	
Fabp1-F	ATGAACTTCTCCGGCAAGTACC	
Fabp1-R	CTGACACCCCCTTGATGTCC	

Cd36-F	ATGGGCTGTGATCGGAACTG	
Cd36-R	TTTGCCACGTCATCTGGGTTT	
Tnfa-F	CCCTCACACTCAGATCATCTTCT	
Tnfa-R	GCTACGACGTGGGCTACAG	
F4/80 (Adgre)-F	ACCAATATCCTGGGGAGAGC	
F4/80 (Adgre)-R	TAGGGCAGATCCCAATTGTC	
Smurf1-F	AGCATCAAGATCCGTCTGACA	
Smurf1-R	CCAGAGCCGTCCACAACAAT	
Smurf2-F	AAACAGTTGCTTGGGAAGTCA	
Smurf2-R	TGCTCAACACAGAAGGTATGGT	
Pparg1-pro-F	ACGCGGAAGAAGAGACCTG	ChIP-qPCR
Pparg1-pro-R	CAGAGTGTGACTTCTCCTCAGC	
Pparg2-pro-F	CTCCTGTTGACCCAGAGCAT	
Pparg2-pro-R	AACATGCAATTTCACCCACA	
Fabp1-pro-F	TCGTTGACCATTGCTCTCAG	
Fabp1-pro-R	GCTTCCTTTCCACAGCTGAC	
